# Microenvironment-triggered multimodal precision diagnostics

**DOI:** 10.1038/s41563-021-01042-y

**Published:** 2021-07-15

**Authors:** Liangliang Hao, Nazanin Rohani, Renee T. Zhao, Emilia M. Pulver, Howard Mak, Olivia J. Kelada, Henry Ko, Heather E. Fleming, Frank B. Gertler, Sangeeta N. Bhatia

**Affiliations:** 1 Koch Institute for Integrative Cancer Research, Massachusetts Institute of Technology, Cambridge, MA 02139, USA; 2 Institute for Medical Engineering and Science, Massachusetts Institute of Technology, Cambridge, MA 02139, USA; 3 Department of Biology, Massachusetts Institute of Technology, Cambridge, MA 02139, USA; 4 Preclinical Imaging, PerkinElmer Inc., Hopkinton, MA 01748, USA; 5 Department of Electrical Engineering and Computer Science, Massachusetts Institute of Technology, Cambridge, MA 02139, USA; 6 Department of Medicine, Brigham and Women’s Hospital and Harvard Medical School, Boston, MA 02115, USA; 7 Broad Institute of Massachusetts Institute of Technology and Harvard, Cambridge, MA 02139, USA; 8 Howard Hughes Medical Institute, Cambridge, MA 02139, USA

## Abstract

Therapeutic outcomes in oncology may be aided by precision diagnostics that offer early detection, localization, and the opportunity to monitor response to therapy. Herein we report a multimodal nanosensor engineered to target tumors via acidosis, respond to proteases in the microenvironment to release urinary reporters, and (optionally) carry PET probes to enable localization of primary and metastatic cancer in mouse models of colorectal cancer. We present a paradigm wherein this multimodal sensor can be employed longitudinally to noninvasively assess burden of disease including tumor progression and response to chemotherapy. Specifically, we showed acidosis-mediated tumor insertion enhanced on-target release of MMP-responsive reporters in the urine. Subsequent on-demand loading of the radio-tracer ^64^Cu allowed pH-dependent tumor visualization, enabling enriched microenvironmental characterization when compared to the conventional metabolic tracer ^18^F-Fluorodeoxyglucose (^18^F-FDG). Through tailored target specificities, this modular platform has the capacity to be engineered as a pan-cancer test that may guide treatment decisions for numerous tumor types.

Precision diagnostics that provide actionable information on the presence, progression, and treatment response of disease are essential for improving oncology outcomes.^1^ Current diagnostic strategies rely on a combination of imaging tests and molecular diagnostic assays to monitor endogenous biomarkers.^2,3^ Imaging tests, such as computed tomography (CT) for lung cancer, remain the clinical standard for detection and localization of disease; however, these methods suffer from poor specificity when employed in a screening paradigm, and can lead to invasive follow-up procedures with risk of complications.^4^ Recent advances in molecular diagnostics have yielded promising assays to measure alternate endogenous disease biomarkers, including blood tests that detect somatic mutations (‘liquid biopsy’) and multi-analyte ctDNA and protein biomarkers (‘CancerSEEK’), that have the potential to identify tissue of origin.^5–7^ Although molecular characterization of mutational heterogeneity offers the capacity for high specificity, strategies that detect circulating endogenous biomarkers face intrinsic sensitivity limitations for early-stage disease and necessitate diagnostic imaging to confirm and localize the disease in a prospective manner.^8–11^ Recognizing that surgery can be curative for many localized solid tumors, a push to develop multimodal tools that couple sensitive, early cancer detection with specific localization is emerging to inform cancer care.^12,13^

To overcome limitations faced by conventional imaging tests and endogenous biomarkers, and with an eye towards designing a future pan-cancer detection tool, we engineered a multi-modal material within the new paradigm of synthetic biomarkers for molecular diagnostics: to induce a signal that did not previously exist in the body.^8,14–16^ These exogenously-administered synthetic biomarkers are selectively activated in the tumor microenvironment to shed reporters into biofluids, and have been shown previously to achieve high-sensitivity detection of tumors *in vivo*.^9,16–18^ As the synthetic biomarker field matures, the focus is shifting to determine how to use these tests to intervene in cancer progression and treatment responses. To close the gap on their clinic actionability, herein we engineered protease responsive imaging sensors for malignancy (PRISM), to combine the delivery of activity-driven synthetic biomarkers with the capacity to be ‘loaded on-demand’ to become a positron emission tomography-computed tomography (PET-CT) imaging agent for both *in situ* and noninvasive disease visualization and monitoring.

This modular material platform leverages co-existing, generalizable hallmarks of invasive tumors - aberrant proteolytic activity and increased extracellular acidification at the invasive tumor front - to enhance the specificity of its diagnostic signal.^19,20^ We exploited a pan-cancer molecular signature, MMP9 that turns on the angiogenic switch and has previously been validated to be more sensitive than cfDNA in mouse models.^9,17,21^ Positive MMP9-derived urinary tests were sequentially confirmed by a diagnostic PET-CT, which precisely localizes the tumor nodules in a way that a molecular predictor of tissue-of-origin cannot.^3,6^ We benchmarked the tumor acidosis-mediated imaging against a conventional metabolic tracer, and demonstrated longitudinal monitoring of cancer progression and regression in two disseminated colorectal cancer (CRC) models treated with a first-line clinical chemotherapy regimen.^22^ Providing both tools in a single modality shortens the regulatory path to patients, and yields an actionable resource with the capacity to inform clinicians on their next interventional steps during monitoring for drug response or relapse. In addition to CRC, the PRISM platform is positioned to be refined as a precision diagnostic for other cancer types by exploiting enzymatic signatures in a range of disease microenvironments, and across a diverse set of oncogenic drivers.

## Nanosensors localize to invasive CRC via tumor acidosis

**Figure 1.**
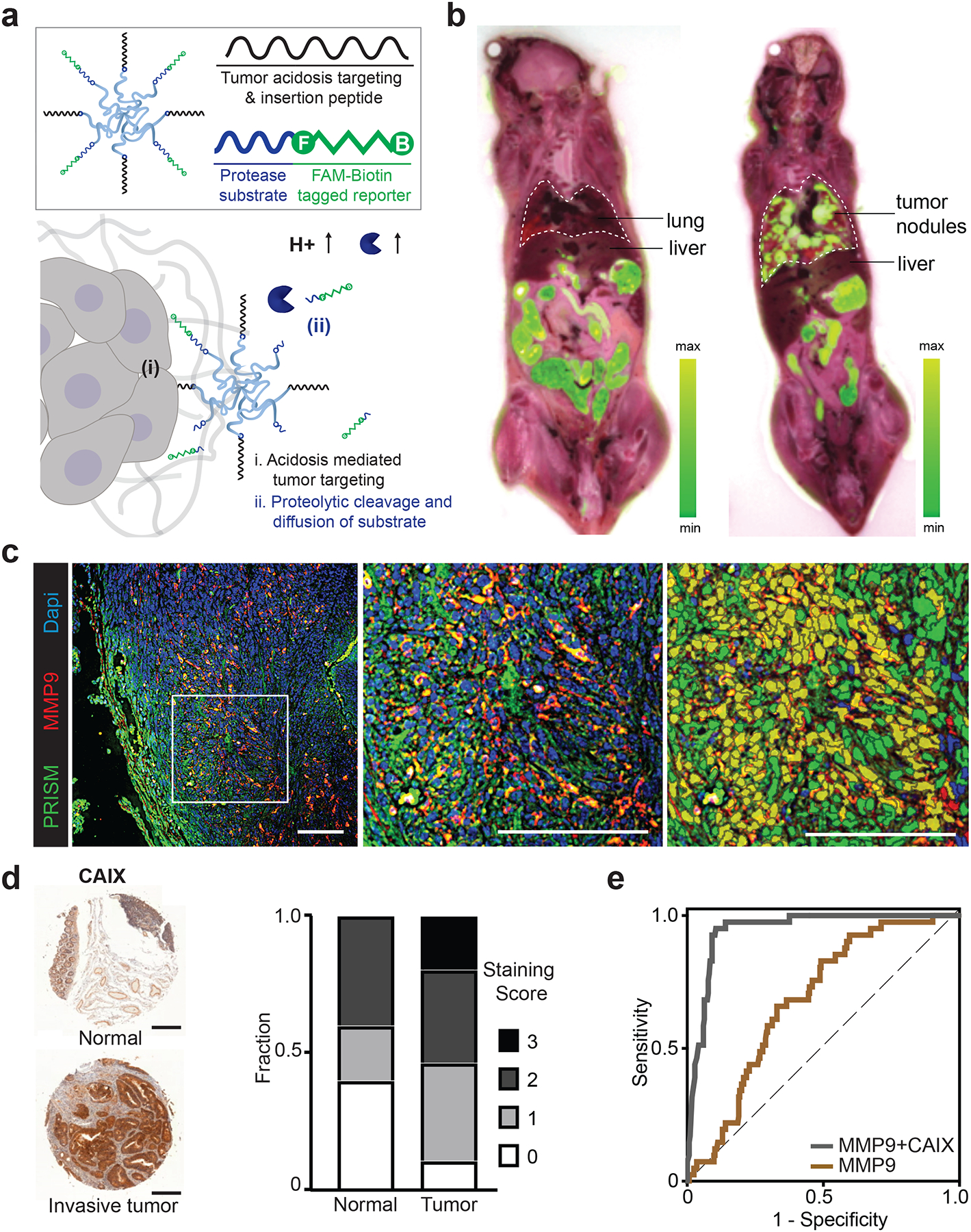
Targeting multivalent nanosensors to invasive CRC via tumor acidosis. **a**, Design of PRISM that targets cancer cells in the acidic TME. (i) Acidosis-mediated tumor insertion enables cancer-specific targeting and imaging. (ii) Activation of PRISM by tumor-specific protease activity triggers release of a synthetic reporter that can partition into urine for noninvasive detection. **b**, Cryo-fluorescence tomography of healthy BALB/c mouse (left) and mouse with lung tumors (right) 6 h after PRISM administration. Images represent individual 25 μm sections from the cryo-macrotome and depict the overlay of bright-and-white organ sectioning with FAM fluorescence from PRISM in the tissue sections. **c**, Colocalization analysis of MMP9 immunofluorescence (red) and PRISM-labeled acidic areas (green) in tumor lesions of lung sections. Left, immunofluorescence staining of a typical tumor-bearing lung section; middle, zoom-in view of the left image; right, cell-based segmentation analysis of the middle image. Number of MMP9-positive and PRISM-positive cells were quantified in three sections. Cell based overlap and Pearson correlation coefficient analysis of 1276 cells indicated 83.1% of MMP9-positive cells overlapped with FAM-staining of the PRISM-positive cells in the tumor. Scale bar= 100 μm. Immunofluorescence staining was completed independently three times by different investigators with similar results. **d**, Representative IHC staining and blind expression scores of CAIX protein levels in patient tissue microarray of normal colon and colon adenocarcinoma (COAD) biopsies. Positive stains are shown in brown. Scale bar= 500 μm. Full TMA staining is shown in Supplementary Fig. 5. Staining was completed twice with similar results. **e**, ROC curves constructed based on mRNA level of MMP9 solely (AUC: 0.683) or dual analytes (MMP9 and CAIX; AUC: 0.946) showing cancer classification as distinct from normal tissue. Dotted line represents a purely random diagnostic tool with an AUC value of 0.5.

The tumor microenvironment (TME) plays an essential role in promoting local invasion and distant metastasis across many types of cancer. On this basis, we elected to engineer a versatile multimodal nanosensor that leverages tumor-specific extracellular molecular activities and, in so doing, develop a single tool for use in both precision diagnosis and treatment monitoring. For the primary function, we harnessed the presence of acidosis in the TME using a pH low insertion peptide (pHLIP) onto a polychain polyethylene glycol (8-arm PEG 40 kDa) polymeric scaffold by click chemistry. This peptide was designed to enable active tumor trafficking of the nanosensor, as pHLIP-mediated extravasation and intracellular insertion are triggered by peptide conformational switch upon acidification in both primary tumors and metastatic lesions (Fig. 1a, Supplementary Fig. 1, 2 & Table 1).^23^ Such membrane affinity and the cooperativity of the transition to the membrane-inserted state can be enhanced via increase of the valency of the peptide display, and inhibited by modification of negatively-charged residues (Asp or Glu) (Supplementary Fig. 1).^24^ To track the nanosensor trafficking to disseminated tumors via a pH switch, we established a transplantation CRC model by intravenous injection of a metastatic murine CRC line (MC26-LucF) in female BALB/c mice.^17,25,26^ Three weeks after tumor inoculation, we performed high throughput cryo-fluorescence tomography on whole animals after systemic administration of FAM-labeled PRISM, and observed the localization of fluorescent signal in a pattern that was perfectly aligned with corresponding anatomical tumors that had formed throughout the lung (Fig. 1b, Supplementary Fig. 3a, b). Using conventional tumor section immunofluorescence, CRC lung nodules exhibited PRISM localization concentrated at the tumor periphery, and colocalized with expression of a key ECM regulator, MMP9 (Fig. 1c). Greater than 80% of detected MMP9 protein expression overlapped with FAM-staining that marks PRISM in the tumor (Fig. 1c, Supplementary Fig. 3c, d).

Microenvironmental acidification is thought to regulate the metastatic potential of tumor and stromal cells through alteration of metabolic reprogramming, angiogenesis, immune suppression, and therapeutic resistance.^27–30^ This effect may be in part due to acidosis-driven signals that enhance the expression of proteases, the activity of which breakdown the ECM and promote tumor invasion.^31^ Cancer Genome Atlas (TCGA) dataset queries revealed that Carbonic Anhydrase 9 (CAIX), a major proton transporter that contributes to extracellular acidification,^31–33^ was overexpressed in human tumors from many tissues (Supplementary Fig. 4a). Staining of CRC tissue microarray (TMA) samples revealed a consistent elevation of CAIX protein expression in tumor sections relative to normal human tissue (Fig. 1d, Supplementary Fig. 5a, c). Consistent with what our group has observed in lung, prostate, and ovarian tumors,^17,18^ analysis of the transcriptomic patterns of two carcinomas (BRCA, COAD) and one blastoma (GBM) revealed several collagen-degrading matrix metalloproteinases (MMPs) as well as ECM components that stimulate cancer cell invasion were also upregulated in CRC (Supplementary Fig. 4b). Notably, when this human sample data was used to test the hypothesis that gene expression could be predictive in disease detection using a receiver operating characteristic (ROC) curve analysis, we observed that while MMP9 expression was moderately diagnostic (AUC=0.683), the combination of MMP9 with CAIX could strongly distinguish tumor samples from controls (AUC=0.946) (Fig. 1e).

In light of the diagnostic power enhancement by combining two ubiquitous hallmarks of cancer, the second TME-based addition to PRISM was an engineered peptide substrate that samples CRC-associated MMP9 activity and releases ligand-encoded reporters into the urine through size-specific kidney concentration. The proteolytic signature of MMP9 turns on the angiogenic switch, even at the earliest stages of tumorigenesis. Elevated MMP9 levels were validated in CRC mouse models and human COAD biopsies (Supplementary Fig. 5b–d). We first tested cleavage kinetics of several reported MMP substrates, and selected one (PLGVRGK) with optimized MMP9 specificity using FRET-based fluorometric peptide substrate cleavage assays with purified MMP9 enzyme and lysates from tumor tissue samples (Supplementary Fig. 6). We then functionalized affinity-tagged, MMP9-responsive peptide onto one polymeric core with the pHLIP peptide or its non-targeting counterpart (Supplementary Fig. 7a). The resulting PRISM or control sensors showed uniform spherical shape and similar sizes (23.93 ± 3 nm and 35.9 ± 10 nm) in transmission electron cryomicroscopy (Cryo-TEM) (Supplementary Fig. 7b, c). Both types of sensors tolerated the click conjugation of the azide-terminated MMP9-activated reporter, pHLIP (or its non-targeting control peptide) and PEG with dibenzocycolctyne (DBCO), yielding monodisperse nanoparticles with equal stoichiometric loading (Supplementary Fig. 7d–f). Conjugation of PEG caused an increase in the hydrodynamic diameter (Supplementary Fig. 7e). When incubated with multiple human and murine cancer cells at acidic pH (6.5) *in vitro*, PRISM exhibited significantly higher cellular accumulation than at physiological pH (7.4) (Supplementary Fig. 8).

## Multimodal monitoring of lung metastases with PRISM

**Figure 2.**
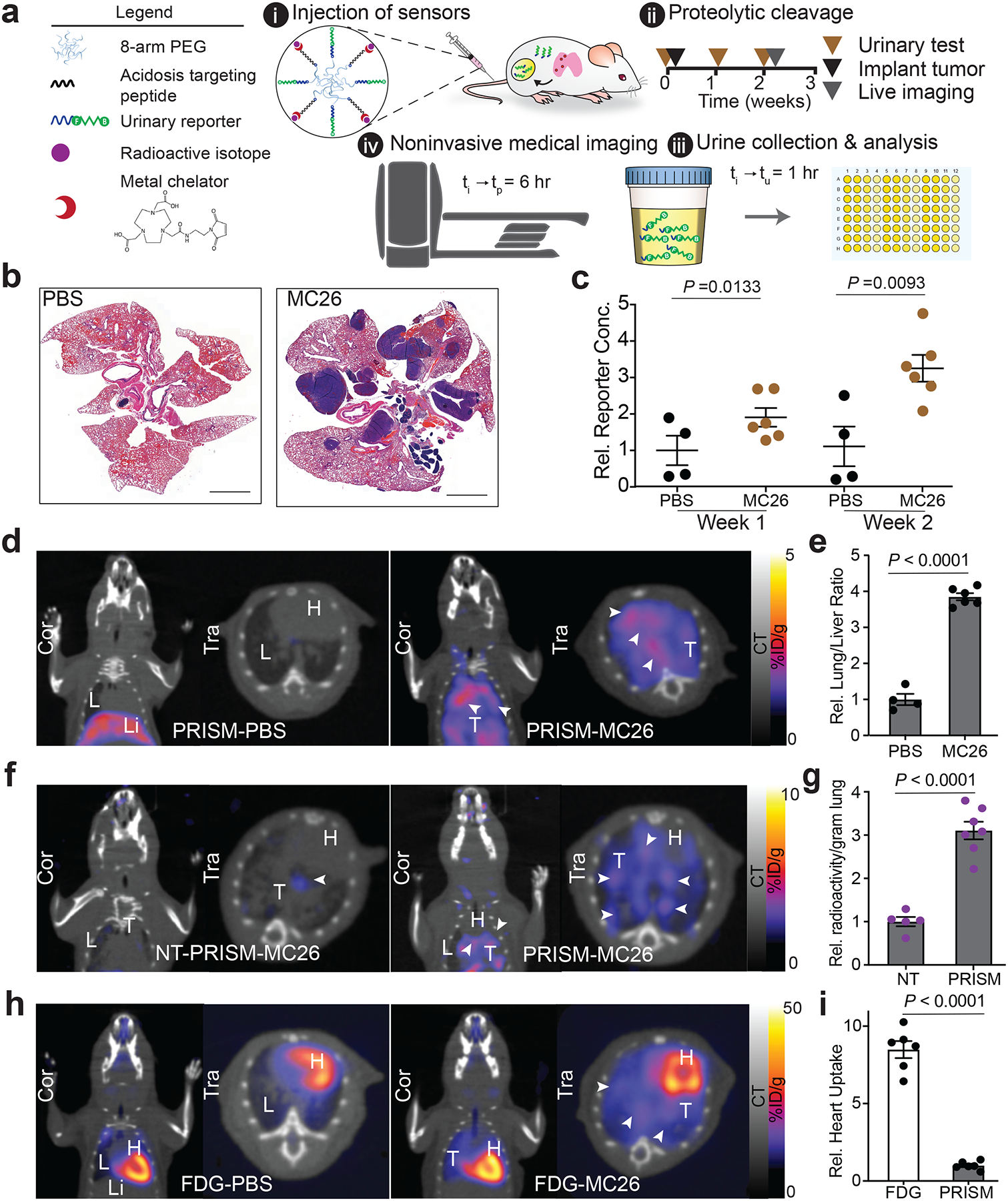
Positron-emitting radionuclide loaded ^64^Cu-PRISM achieved multimodal monitoring of CRC lung nodules. **a**, Schematic showing functional moieties, i.e. tumor targeting peptide, protease substrate, imaging tracers displayed on a nanosensor surface (i), study time course of tumor transplantation (ii), urinary testing (iii), and PET-CT imaging (iv) in a syngeneic CRC pulmonary disease model. **b**, Representative histological images of lungs from healthy and CRC tumor-bearing BALB/c mice on day 17 after tumor inoculation. Scale bar= 2 mm. Experiments were completed independently three times with similar results. **c**, Relative reporter concentrations measured in the urine of healthy mice (n=4 mice per group) vs. CRC lung tumor-bearing mice (n=6 mice per group) after application of PRISM (data are represented as means ± SEM; unpaired two-tailed *t*-test with Welch’s correction). **d**, PET-CT images of healthy and tumor-bearing mouse lungs at week 2, 6 h after application of ^64^Cu-PRISM, coronal view (Cor) and transverse view (Tra). Arrow, presence of CRC lung tumors. L, lung; Li, liver; H, heart; T, tumor nodules. **e**, Quantification of representative images in (**d**) showing elevated lung-to-liver ratio in lung tumor-bearing mice (n= 6 mice per group) compared with healthy control mice (n=4 mice per group, data are shown as means ± SEM; normalized to the healthy control animal; unpaired two-tailed *t*-test with Welch’s correction). **f**, Representative PET-CT images of the lung in animals imaged with ^64^Cu-non-targeting (NT)-PRISM (left panel) or ^64^Cu-PRISM (right panel). Arrowheads mark the presence of CRC lung tumors. L, lung; H, heart; T, tumor nodules. **g**, *Ex vivo* quantification of the radioactivity accumulated in the lungs extracted from animals imaged with ^64^Cu-NT-PRISM (n= 5 mice per group) or ^64^Cu-PRISM (n= 7 mice per group), represented as ^64^Cu counts per minute/gram tissue (data are represented as means ± SEM; unpaired two-tailed *t*-test with Welch’s correction). **h**,^18^F-FDG PET-CT scans of the same healthy and tumor-bearing mice in (**d**) 1 h after tracer injection showing signal accumulation from active glucose uptake. L, lung; Li, liver; H, heart; T, tumor nodules. **i**, Quantification of images in (**d**) and (**h**) indicating a significantly lower background in the chest when lung tumor-bearing animals are imaged with PRISM compared with conventional ^18^F-FDG tracer (data are shown as means ± SEM; normalized to the tumor-bearing animal imaged with PRISM, unpaired two-tailed *t*-test with Welch’s correction).

After characterizing the TME-directed nanosensors, we assayed whether they could detect tumor lesions that are notoriously challenging to detect: lung tumors.^34^ In conventional medical imaging such as PET, lung tumor signals are often masked by the dramatic uptake of the glucose analog ^18^F-2-deoxyfluoroglucose (^18^F-FDG) by metabolically-active cardiac cells.^35^ Furthermore, the false positive detection rate of clinical lung masses is high, due to the prevalence of accumulated scar tissue and other benign structures that can appear indistinguishable from malignant and growing lung lesions. To enable PET-based imaging via isotope-loaded PRISM, we site-specifically labeled pHLIP with the metal chelator 1,4,7-triazacyclononane-triacetic acid (NOTA) molecule to bind the positron-emitting radionuclide (^64^Cu, *t*_1/2_= 12.701 ± 0.002 h) (Fig. 2a, Supplementary Fig. 2). Based on the discriminatory power of targeting tumor acidosis (Fig. 1b, Supplementary Fig. 3a, 8), we hypothesized that PRISM could optimize PET signal by promoting longer *in vivo* circulation times and enhanced tumor-homing through multivalent orientation of pHLIP. We then validated efficacy of the ^64^Cu-PRISM in the aforementioned CRC lung tumor model, in which lung nodule formation was observed within 2–3 weeks of systemic inoculation of tumor cells (Fig. 2b).

Upon intravenous administration of nanosensors, urine samples from tumor-bearing and healthy control mice were collected after 1 h, and the ELISA readouts of protease-liberated reporter levels increased as tumor growth progressed (Fig. 2a, c). *Ex vivo* fluorescence scanning, histological and Immunofluorescence analyses of PRISM distribution in tumor-bearing and healthy lung tissues revealed tumor-specific sensor accumulation, suggesting that the pH-selective targeting of PRISM via pHLIP enhanced on-target MMP9 cleavage (Supplementary Fig. 9a, c–e; Supplementary Fig. 10). Furthermore, in the same mice that exhibited significant elevation of the MMP-dependent urine signal at the week 2 time point, PET-CT detection using ^64^Cu-loaded PRISM localized CRC lung tumors, resulting in substantially increased positive-to-negative tumor ratios (3.85 ± 0.25) (Fig. 2d, e, Supplementary Table 2). Consistent with the fluorescent readout (Supplementary Fig. 10), such tumor-specific PET signal was not observed in animals injected with non-acidosis-targeting control nanosensors, as verified by 3.11 ± 0.23-fold tracer accumulation in the tumor-bearing lungs exposed to ^64^Cu-PRISM, relative to the signal derived from the control sensor (Fig. 2 f, g). Importantly, we re-imaged the same mice shown in Figure 2d using PET-CT with the conventional PET tracer used in clinics, ^18^F-FDG, and compared the results with those obtained via imaging with ^64^Cu-PRISM. Although the standard uptake value remained comparable when imaging the lung, ^18^F-FDG exhibited a strong accumulation in the heart, and the resulting high background signal obscures some of the lung-derived signal that is visible in ^64^Cu-PRISM-derived images, which gave rise to significantly less background signal in the chest (Fig. 2h, i). Notably, we observe no adverse effects in the kidney or liver following repeated administration of the PRISM nanosensors in these immunocompetent hosts, as reflected by the normal histological staining and clearance kinetics (Supplementary Fig. 11). Therefore, PRISM offers great potential in detecting tumors in the lung.

## Longitudinal, multimodal monitoring of CRC liver nodules

**Figure 3.**
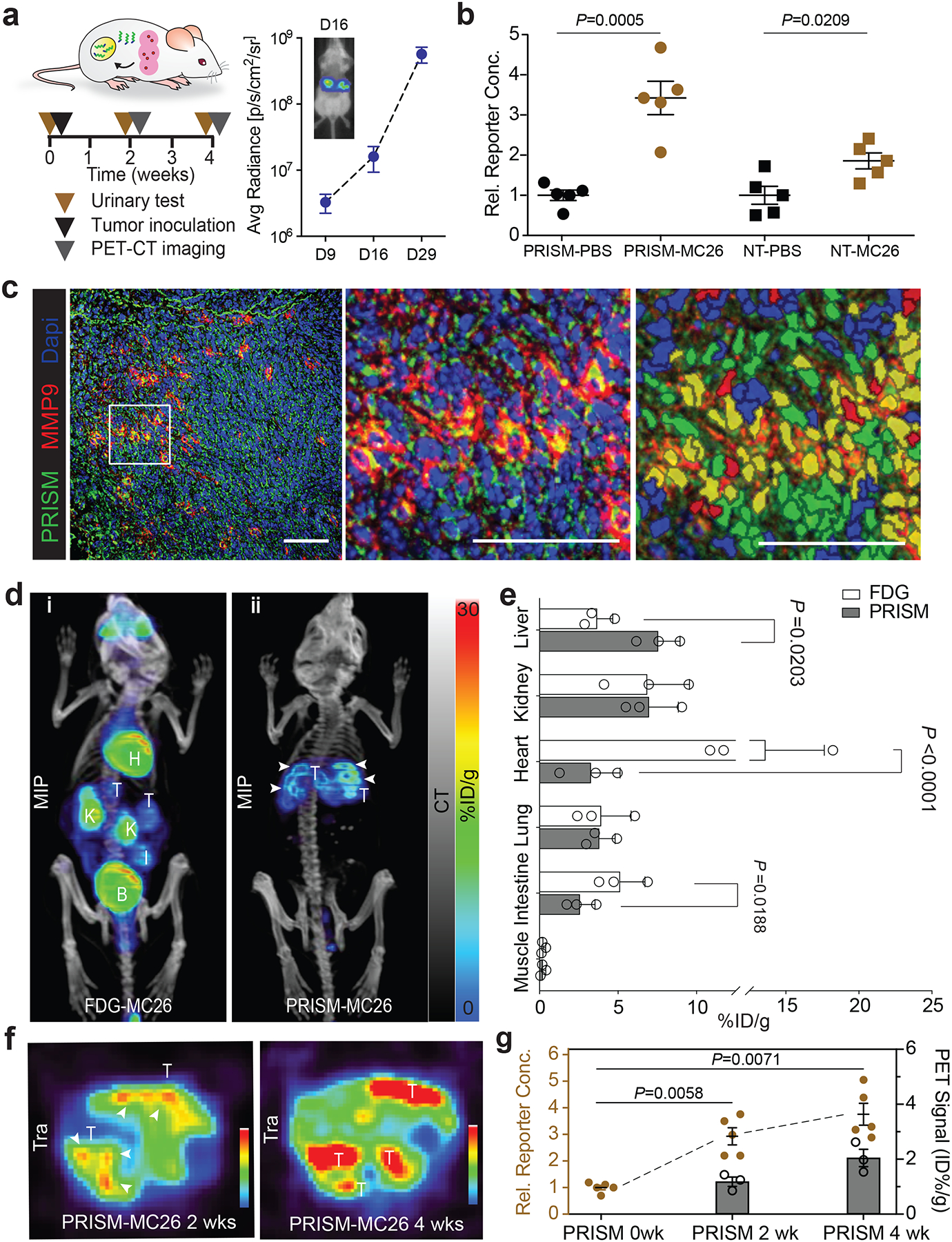
PRISM enabled longitudinal, multimodal monitoring and imaging of CRC liver nodules. **a**, Schematic showing PRISM carrying radioactive PET-CT tracer tested in a syngeneic model of CRC hepatic invasion for urinary detection and imaging capabilities, as well as study time course of tumor transplantation, urinary testing and PET-CT imaging. Liver tumor formation was confirmed by bioluminescence imaging of tumor-bearing mice over time (n=10 mice, data are shown as means ± SEM). **b**, Relative reporter concentrations in the urine 1 h after PRISM and its untargeted counterpart application (n=5 mice per group, data are represented as means ± SEM; unpaired two-tailed *t*-test with Welch’s correction). **c**, Colocalization analysis of MMP9 immunofluorescence (red) and PRISM-labeled acidic areas (green) in tumor lesions of liver sections. Left, immunofluorescence staining of a typical tumor-bearing liver section; middle, zoom-in view of the left image; right, cell-based segmentation analysis of the middle image. Number of MMP9-positive and PRISM-positive cells were quantified in three sections. Within the 1130 cells quantified, 60.4% of MMP9 positive cells overlapped with PRISM-localized cells in the tumor (Supplementary Fig. 12e). Scale bar= 100 μm. **d**, Representative maximum intensity projection (MIP) of CRC liver tumor-bearing mice following administration of ^18^F-FDG (i) and ^64^Cu-PRISM (ii) two days apart. **e**, Quantitative analysis of the key organs including liver, kidney, heart, lung, intestine, and muscle in tumor-bearing mice imaged with ^18^F-FDG and ^64^Cu-PRISM during disease progression. (n=3 mice per group, means ± SD; unpaired two-tailed *t*-test with Welch’s correction). **f**, Transverse view of liver nodules in tumor-bearing animals imaged longitudinally at 2 weeks and 4 weeks after tumor inoculation. **g**, Longitudinal, multimodal monitoring of urinary reporter level and positive-to-negative tumor ratios in the liver quantified in PET-CT imaging at 2 weeks and 4 weeks after tumor inoculation. (n=5 mice per group for urinary reporter, means ± SEM, Kolmogorov-Smirnov test for normal distribution followed by Brown-Forsythe and Welch ANOVA test with Dunnett’s T3 multiple comparisons; n=3 mice per group for PET imaging at 2 and 4 weeks post tumor inoculation, means ± SD).

Besides the multimodal monitoring of tumor progression in the lung, we next evaluated the efficacy of the PRISM platform in the liver, another organ that has also been clinically challenging to image due to its elevated background uptake.^36^ Clinically, CRC primarily migrates to liver, and this process can be surgically induced in mice via intrasplenic injection of the CRC cell line. This procedure exhibited robust tumor penetrance, such that 70–80% of immunocompetent BALB/c mice formed liver nodules within two weeks after injection of MC26-LucF, allowing for total burden monitoring via bioluminescence imaging (Fig. 3a, Supplementary Fig. 12a, b). At this timepoint, PRISM particles were systemically administered to both control and tumor-bearing mice and specifically accumulated to CRC nodules spreading in diseased liver (Supplementary Fig. 12c), urine samples were collected 1 h after sensor injection and the protease-liberated reporter levels were detected by ELISA. Consistent with the lung tumor results, urinary signals were significantly elevated in tumor-bearing animals, relative to healthy controls (Fig. 3b). Moreover, when the nanosensors incorporated a non-targeting counterpart of pHLIP, the tumor-specific signal was dampened almost two-fold, suggesting that the pH-selective targeting of PRISM enhanced sensitivity of detection (Fig. 3b). These observations aligned with *in vivo* and *ex vivo* overlap of tumor and pHLIP-containing PRISM but not with the control NT-PRISM (Supplementary Fig. 12c, d). The tumors were allowed to progress for an additional two weeks, after which ^64^Cu-PRISM were re-administered, and following both urine testing and live imaging, the mice were sacrificed, and livers were subjected to *ex vivo* immunofluorescent histochemistry. MMP9 staining was elevated in liver tumors, and largely colocalized with FAM-staining that marks PRISM probes (Fig. 3c). Quantitatively, greater than 60% of MMP9 expression overlapped with PRISM-positive cells in acidic areas close to the tumor-stroma interface, where hallmarks of tumor invasion are enriched (Fig. 3c, Supplementary Fig.12e).

In addition to the urine-based detection via PRISM, we evaluated whether PRISM could track tumor progression using PET-CT imaging. In tumor-bearing animals two weeks post-splenectomy, PET-CT imaging at 6 h post-intravenous injection of ^64^Cu-PRISM (and 5 h post urine collection) showed bright illumination of CRC liver tumor that particularly enriched at the invasive front at the tumor-stroma interfaces. Tissue uptake of ^64^Cu-PRISM was measured in tumors and surrounding liver tissue, resulting in positive-to-negative tumor ratios of 2.2:1 (Fig. 3d, f, g). We also found that the standard uptake value in major organs such as liver, kidney, lung, intestine was comparable between the ^64^Cu-PRISM and ^18^F-FDG (Fig. 3e). Furthermore, we observed similar signal intensity in MC26 tumor versus liver ratios in the same mice imaged with either ^18^F-FDG and ^64^Cu-PRISM (Fig. 3e), suggesting that ^64^Cu-PRISM are at least as sensitive as conventional reagents. In addition, when PRISM was used to perform longitudinal tracking of tumor progression in individual animals, the ^64^Cu-PRISM PET measurement of liver tumor expansion (Fig. 3f) matched a corresponding increase in MMP9-cleavable reporter signals over time (Fig. 3g). An important clinical limitation of ^18^F-FDG-based PET imaging is its dependence on high glucose uptake by the target cells. In contrast, tumors derived from the human CRC line with low glucose uptake (LS 174T)^37^ demonstrated an 8.6-fold tracer uptake over the surrounding normal muscles when ^64^Cu-PRISM was used, whereas the tumor uptake of ^18^F-FDG was indistinguishable from the background tissue (Supplementary Fig. 13).

## Noninvasive assessment of tumor response to therapy

**Figure 4.**
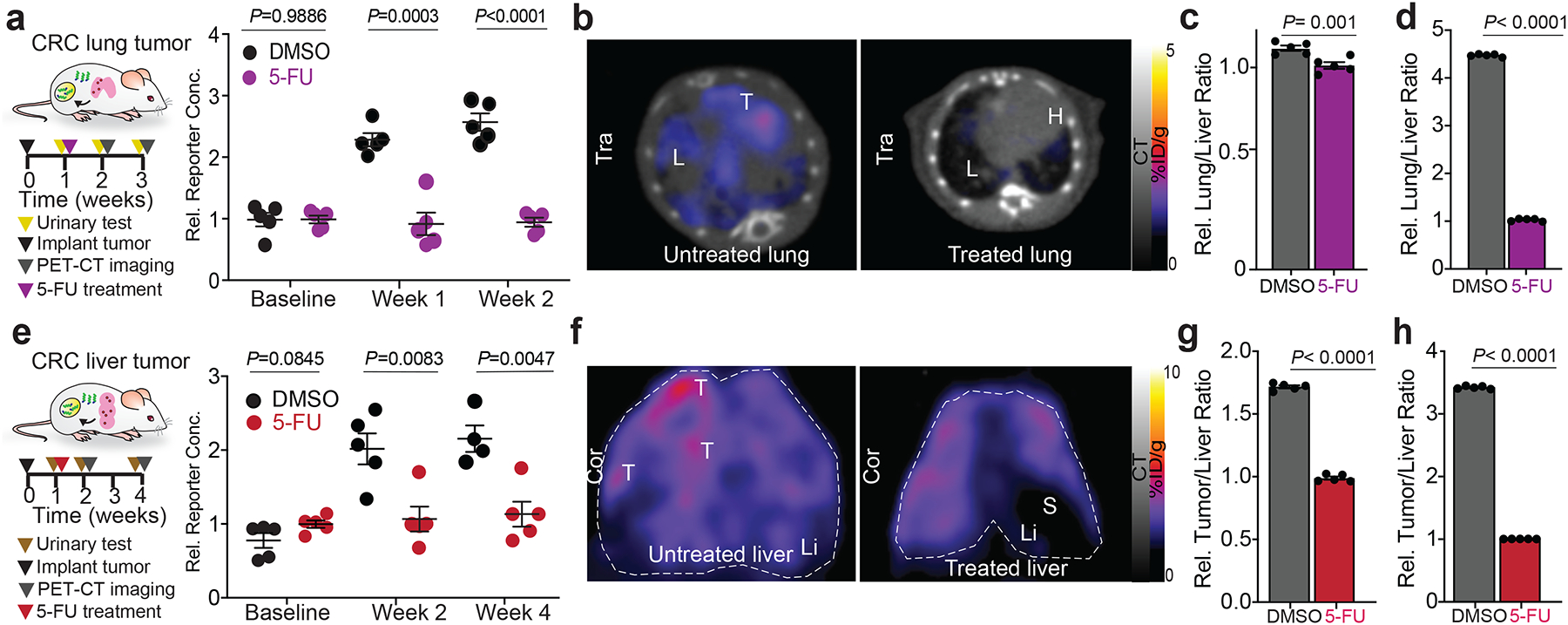
Noninvasive multimodal monitoring of therapeutic response to first line chemotherapeutic by PRISM. **a**, Schematic of drug treatment monitoring testing in the aforementioned CRC lung metastasis model. Two categories of mice were grouped: one group received the vehicle control (DMSO) in PBS; the other received 5-FU at 15 mg/kg in PBS. The mice were chosen to balance a similar range of tumor burden between the groups. Relative reporter concentrations measured in the urine of lung tumor-bearing mice with or without 5-FU treatment (n=5 mice per group; data are represented as means ± SEM; mean normalized to 5-FU treated mice; unpaired two-tailed, *t*-test with Welch’s correction; not significant, *P*=0.9886 for baseline). **b**, PET-CT transverse view (Tra) of tumor progression after 2 weeks of 5-FU treatment in mice with CRC lung tumors. Relative lung-to-liver ratio quantified from longitudinal PET-CT images collected after one (**c**) or two weeks (**d**) of 5-FU treatment in mice with CRC lung tumors (Data are shown as means ± SEM; mean normalized to 5-FU treated mice; unpaired two-tailed, *t*-test with Welch’s correction). **e**, Schematic of drug treatment monitoring testing in the CRC liver metastasis model. The untreated group received the vehicle DMSO in PBS; the treated group received 5-FU at 15 mg/kg in PBS. Relative reporter concentrations measured in the urine of liver tumor-bearing mice with or without 5-FU treatment (n=5 mice per group; data are represented as means ± SEM; mean normalized to 5-FU treated mice; unpaired two-tailed *t*-test with Welch’s correction; not significant, *P*=0.0845 for baseline). **f**, Coronal view (Cor) of PET-CT images of tumor progression after 3 weeks of 5-FU treatment in mice with CRC liver tumors. Relative positive-to-negative tumor ratios in the liver were quantified from longitudinal PET-CT images collected after 1-week (**g**) or 3-week (**h**) of vehicle or drug treatment in untreated and 5-FU treated cohorts (n=5 mice per group, mean normalized to 5-FU treated mice, unpaired two-tailed *t*-test with Welch’s correction).

Although a large proportion of CRC patients experience liver or lung metastases, the predominant intervention used for patients with this diagnosis remains treatment with 5-FU/Leucovorin, a standard chemotherapy. During the course of treatment, noninvasive response monitoring is essential to screen for tumor recurrence after surgical resection, and to derive individualized assessment of drug-target interactions and subsequent efficacy. Therefore, we employed PRISM to monitor the local extracellular activities of tumors during drug treatment. We divided cohorts of either lung or liver CRC tumor-bearing BALB/c mice into experimental groups that received a course of 5-FU treatment, and control groups that received only vehicle (Fig. 4a, e). The disseminated lung tumor model is a more rapidly progressing disease, and so these animals were followed for a total of 2 weeks (Fig. 4a–d), whereas the liver CRC-bearing mice were monitored for 4 weeks (Fig. 4e–h). PRISM sensors were administered to each group at the beginning of 5-FU treatment, and then weekly thereafter. With each administration, urinary reporter concentration was assayed 1 h after intravenous injection of the nanosensors, providing a readout of *in vivo* proteolytic activity, and that we hypothesized to reflect tumor invasion capacity. After an additional 5 h (6 h post-PRISM injection), mice were scanned in a PET-CT imager for visualization of tumor number and size. As anticipated, 5-FU treatment in the CRC lung disease model significantly inhibited tumor progression compared with the non-treated cohort, which was apparent both in the decreased urinary reporter signal (Fig. 4a), and the PET imaging readout (Fig. 4c), even after a single week of intervention. At the 2-week timepoint, untreated mice exhibited substantially increased tumor burden in the lung (Supplementary Fig. 14a, b), and disease had progressed to the point of requiring euthanasia. Quantitatively, the relative lung/liver ratio of PET-CT signal in the cohort that received no treatment was over 3 times higher than that of the cohort treated with chemotherapy for two weeks (Fig. 4b, d). Consistent results were obtained when we applied the same longitudinal monitoring of treated and untreated mice bearing CRC liver metastases (Fig. 4e–h, Supplementary Fig. 14c, d). This robust example of the dual-function, single particle diagnostic PRISM nanosensor demonstrates the potential value of this tool when evaluating the efficacy of new candidate treatments, given the correspondence of the urinary sensor that reads out tumor invasion capacity and the acidosis-dependent imaging of the tumor itself. In other words, with the PRISM platform, we can monitor tumor size and activity in a single injection, which may help discriminate between therapeutic candidates that exhibit more or less effectiveness in both axes at the same time.

## Discussion

We developed a modular approach that leverages disease hallmarks to noninvasively detect cancer, localize malignant lesions for surgical intervention, and longitudinally monitor drug response. In our approach, the remarkable concurrence of acidosis and MMPs across human cancer model systems was leveraged to achieve specific targeting of aggressive tumors. This noninvasive tool arose from the application of synthetic biomarkers to amplify disease-associated protease activity and provide a concentrated urine-based readout. The localization capability arose from acidosis-targeting and loading with ^64^Cu enabling quantitative imaging by PET-CT. The multimodal monitoring of drug response arose from longitudinal assessment of tumor nodules in both the lung and the liver by urinary reporter and PET-CT in response to cytotoxic chemotherapy.

To minimize off-target effects, cancer diagnosis and therapy rely on active targeting strategies that are often limited to specific ligands expressed in diseases. Due to increased fermentative metabolism and poor perfusion, extracellular pH in solid tumors is ubiquitously acidic (pH ~6.5–6.9) compared to normal tissue under physiologic conditions (pH ~7.2–7.4).^19^ Such acidification is dynamic and heterogeneous as tumors progress, and the regions of highest tumor invasion correspond to areas of lowest pH.^27,31,38^ In our models, acidic regions marked by PRISM overlapped with highly invasive regions at the tumor-stroma interface, where increased expression of MMPs was observed (Supplementary Fig. 3d, 12e). Engaging the aggressive population at the invasive front of tumors significantly improved specificity of detection (Fig. 2, 3). Although this work is not centered on the detection sensitivity of activity-based nanosensors, PRISM enables urinary signals to reveal tumors below 2 mm, informed by a pharmacokinetic mathematical model (Supplementary Fig. 15, 16). Such prediction agreed with our previous report on tumor-penetrating ligand-functionalized activity-based nanosensors, that detected nodules with median diameters smaller than 2 mm in an orthotopic model of human ovarian cancer.^17^ In order to access the smallest tumor sizes that PRISM could detect, urinary tests performed shortly after initial transplantation of syngeneic CRC lung tumors showed differential urinary reporter signal just 7 days post-tumorigenesis, when the median histological diameters of disseminated tumor nodules were below 0.1 mm (Supplementary Fig. 17).

Optical, nuclear medicine, and magnetic resonance imaging (MRI) probes can visualize acidic or proteolytic tumor microenvironments.^14,39–41^ PET imaging using non-FDG radiotracers, such as nucleoside, amino acid analogs, antibody and fragments, or polymers shows significant potential for tumor detection in organs with an undesirable metabolic background (i.e. brain, heart, kidneys) or in low FDG uptake tumors.^42–44^ Likewise, tumor-anchoring pHLIP-mediated PET-CT imaging overcame the insufficient tumor contrast via passive accumulation due to leaky tumor vasculature alone (Fig. 2f, g),^44^ but also eliminated background signal in healthy surrounding tissue due to high physiological uptake of FDG (Fig. 2d, e, h, i). PRISM detected multiple cancer types *via* the broad presence of tumor acidosis (Supplementary Fig. 18), particularly in low-glucose-metabolizing colon tumors (Supplementary Fig. 13), shedding light on its use for diagnosing FDG-insensitive malignancies such as low-grade lung adenocarcinoma or mucinous neoplasms. Compared to PET imaging with radiolabeled pHLIP only, polymerization of PRISM extended circulatory half-life and enhanced target recognition through multivalency (Supplementary Fig. 9b). Although PEGylation reduces accumulation in kidneys, high uptake of pHLIP in the naturally acidic regions (i.e. renal cortical interstitium) may preclude the use of this agent for disease detection in these sites (i.e. renal cell carcinoma).^45,46^

Despite its clinical utility in tumor diagnosis and staging, PET-CT with^18^F-FDG has a limited role in widespread primary cancer screening because of relatively low disease prevalence. Thus, conducting PET-CT in patients with positive molecular diagnostic results would significantly improve its performance. We envision that the noninvasive, PRISM-based urine readout could be applicable for at-risk patients, identified either due to risk factors or as a preventative monitoring following surgical tumor resection, offering an affordable and less time-intensive screening. When tumor occurrence is indicated by a longitudinal change in urinary signal, the quantitative signal derived from PET-CT imaging can be employed to confirm and localize tumor burden using the same core PRISM materials after loading them with a radiotracer on demand, thus eliminating unnecessary exposure to radioactivity. Therefore, beyond contributing to an initial diagnosis, PRISM can also be used in patient stratification leading to surgery with an intent to cure. This tool may also reveal regionally-localized tumor responses, or non-responses, providing clinicians with even more robust and nuanced data with which to adjust treatment paradigms in a patient-specific manner. In the long run, we anticipate that pan-cancer tests will likely emerge that allow clinicians to assess patients for many cancers simultaneously at an annual check-up through a combination of molecular diagnostics. The principle of combining screening tests with on-demand diagnostic imaging may be incorporated into routine medical care, the feasibility of which has been encouraged in an exploratory trial on multi-cancer blood testing combined with PET-CT.^3^

The PRISM platform represents a step towards establishing clinical actionability of molecular diagnostics in a single entity, but further *in vivo* validation is needed to confirm its generalizability beyond gastrointestinal cancers. The versatile sensor design enables detection of different extracellular hallmarks and development of probes for variable imaging modalities such as near-infrared optical imaging, MRI, or PET/MRI, depending on the disease location, anatomy, and biology. Owing to the heterogeneity and benign disease etiologies in human cancers, clinical trials will be necessary to accurately validate the clinical utility of such material, with the ultimate goal of improving the effectiveness of precision diagnostics.

## Methods

### Peptide & peptide conjugate synthesis and characterization.

Information of all peptides used in this study was listed in Supplementary Table 1. Peptides were synthesized by CPC Scientific, Inc. unless otherwise indicated. All commercially synthesized peptides were purified by the vendor using reversed-phase high-performance liquid chromatography (RP-HPLC) on a Phenomenex Luna C18 column to > 90% purity. The molecular weight of purified peptides was verified by Mass Spectral Analysis. One of the variants of pH low insertion peptide (pHLIP-V3) with improved imaging capacity was utilized in the study,^23^ the peptide was stabilized with two lysine residues at the C-terminus to improve its solubility and purity in solid phase synthesis. Peptide conjugates were made by covalently conjugating maleimide-containing fluorophores or the metal chelator NOTA (1,4,7-triazacyclononane-N,N’,N”-triacetic acid) (Macrocyclics Inc., B-622) to the single cysteine residue of the peptide. To label pHLIP-V3, peptide was dissolved in N,N-Dimethylformamide (DMF)/H_2_O (1:4) (Sigma-Aldrich) at 2 mg/ml followed by slow addition of 5 eq. of maleimide-containing fluorophore or chelator in equal volume of phosphate buffer (0.1 M, pH 7.0). The reaction was conducted at room temperature for at least 4 h. Similarly, the non-targeting control peptide was dissolved at 1 mg/ml in H_2_O and reacted with 5 eq. of fluorophore or chelator at room temperature for at least 4 h. Resulting peptide conjugates were washed three times by Amicon^®^ Centrifugal Filter Units (MWCO 3 kDa, MilliporeSigma) and analyzed on an Agilent Model 1100 HPLC system with a Vydac^®^ 214TP510 C4 column using the ChemStation software. The HPLC gradient was 5% to 5% A buffer (0.05% TFA in H_2_O) isocratic for 20 min, and to 80% B buffer (0.05% TFA, 99.95% ACN) at 65 min with a flow rate of 0.3 mL/min. Purified conjugates were validated by MALDI-TOF mass spectrometry (Microflex MALDI-TOF, Bruker Co.) using α-Cyano-4-hydroxycinnamic acid matrix. To screen MMP9 substrate with optimal signal-to-background ratio, intramolecular-quenched peptides were used in fluorometric protease cleavage assays. The peptide substrate sequence selected was tagged with D-stereoisomer of glutamate fibrinopeptide, flanked by a Biotin/FAM ligand as the urinary reporter for *in vivo* experiments.

### Nanosensor synthesis and characterization.

Multivalent PEG with reactive groups (40 kDa, eight-arm, JenKem Technology) was dissolved in Chelex 100 Resin (Bio-Rad Laboratories, Inc) processed phosphate buffer (0.1 M, pH 7.0) and filtered (pore size: 0.2 μm). After filtration, an equimolar mixture of azide-containing pHLIP-V3 (or non-targeting control peptide, or peptides conjugated with fluorophore or chelator) and urinary reporter peptides were added at 2-fold molar excess to the DBCO groups on 8-arm PEG (pHLIP-V3 or NT-V3: reporter: DBCO= 1:1:1) and reacted for at least 4 h at room temperature. Nanosensors were washed three times by Amicon^®^ Centrifugal Filter Units (MWCO 10 kDa, MilliporeSigma) to separate from unconjugated peptides and further purified using fast protein liquid chromatography (FPLC, GE Healthcare) with a Superdex 75 10/300GL column. Concentration was quantified by extinction coefficients of ligands. Nanosensors were stored at 4 °C in PBS. Dynamic light scattering (Zeta Sizer Nanoseries, Malvern Instruments, Ltd) was used to characterized the hydrodynamic diameter of the nanosensors. Ratio of pHLIP-V3 or non-targeting control peptide and MMP substrate on sensor was quantified by measuring absorbance of Cy7-labeled pHLIP-V3-F or NT-V3-F (750 nm) and FAM labeled MMP9 substrate (495 nm) on UV-Vis spectrum using a Nanodrop Spectrophotometers (Thermo Scientific).

### Transcriptomic analysis.

RNA-Seq data of two carcinomas (BRCA, COAD) and one blastoma (GBM) were obtained from the Cancer Genome Atlas (TCGA) Research Network (http://cancergenome.nih.gov). Differential gene expression was analyzed at the BioMicro Center at MIT using transcriptomic data from both tumor and control samples. The BRCA datasets contain 1093 tumor samples and 112 normal tissue samples; the COAD datasets involve 285 tumor samples, 41 normal tissue samples; and the GBM datasets include 153 tumor samples, 5 normal tissues samples. Briefly, next generation sequencing reads were mapped to mouse mm10 reference genome using star/2.5.3a. Gene level expression calculation was performed by rsem/1.3.0. Differential expression analyses were carried out by DESeq2 1.10.1. Expression profiles of particular genes of interest among multiple tumor types were downloaded from FireBrowse (http://firebrowse.org).

### Circular dichroism.

Acidosis targeting peptide (V3) and its non-targeting counterpart were dissolved at 1 mg/mL in 20 mM HEPES buffer (pH 7.4) or 20 mM MES (pH=6.0), respectively. Circular dichroism spectra were recorded at 0.2 mg/ml at 25 °C on a JASCO J-1500 model circular dichroism spectrometer. The molar ellipticity was shown based on CD signal and peptide sample concentration.

### Cryogenic transmission electron microscopy (cryo-TEM).

In sample preparation for cryo-electron microscopy, 3 μL of sensors at 0.5–1 mg/mL (concentration was quantified by absorbance of FAM on MMP responsive peptide) and buffer containing solution was dropped on a lacey copper grid coated with a continuous carbon film and blotted to remove excess sample without damaging the carbon layer by Gatan Cryo Plunge III. Grid was mounted on a Gatan 626 single tilt cryo-holder equipped in the TEM column. The specimen and holder tip were cooled down by liquid-nitrogen, which keeps maintaining during transfer into the microscope and subsequent imaging. Imaging on a JEOL 2100 FEG microscope was done using minimum dose method that, were essential to avoid sample damage under the electron beam. The microscope was operated at 200 kV and with a magnification in the ranges of 10,000~60,000 for assessing particle size and distribution. All images were recorded on a Gatan 2kx2k UltraScan CCD camera.

### Cell culture and flow cytometry.

Mouse cell lines MC26-LucF (carrying firefly luciferase, from Kenneth K. Tanabe Laboratory, Massachusetts General Hospital), D8–175 KPC-derived pancreatic cancer line (from Tyler Jacks Laboratory, Massachusetts Institute of Technology) and 4T1 (ATCC CRL-2539); human cell lines MDA-MB-435 (NCI-60) and U937 (ATCC CRL-1593.2) were cultured in DMEM (Gibco) medium supplemented with 10% (v/v) fetal bovine serum (FBS)(Gibco), 1% (v/v) penicillin/streptomycin (CellGro) at 37 °C and in 5% CO_2_. OVCAR8 (NCI-60) and LS 174T (ATCC CL-188) cell lines were grown in RPMI1640 (Gibco) or EMEM (Gibco) medium supplemented with 10% (v/v) FBS and 1% (v/v) penicillin/streptomycin, respectively. All cell lines tested negative for mycoplasma contamination. For the alteration of the pH conditions in the medium for each cell line, the sodium bicarbonate concentration was adjusted based on the Henderson-Hasselbalch equation in the base medium to reach a target pH. Media was equilibrated at 37 °C and 5% CO_2_ for at least 12 h prior to use. For flow cytometry analyses, cells cultured in 6 well plates were treated with 10 μM of fluorescent-labeled pHLIP-nanosensors at physiological (7.4) or acidic pH (6.4). Cells were collected by pipetting with and resuspending in DAPI (0.5 μg/mL)-containing PBS (pH 7.4 or 6.4) and analyzed on a BD^™^ LSRII flow cytometer immediately. Data were analyzed in FlowJo (TreeStar Software).

### Animal models.

All animal studies were approved by the Massachusetts Institute of Technology Committee on Animal Care (MIT protocol 0417-025-20 & 0217-014-20). Animals were maintained in the Koch Cancer Institute animal facility, with a 12-h light/12-h dark cycle, at ~18–23°C and ~50% humidity. All animals received humane care, and all experiments were conducted in compliance with institutional and national guidelines and supervised by Division of Comparative Medicine (DCM) of MIT staff. The CRC lung metastasis model was generated by intravenous injection of 1 × 10^5^ luciferized mouse MC26 cell line in female BALB/c mice (6–8 weeks, Taconic). To generate a liver metastasis model of CRC, immunocompetent, 6–8-week-old female BALB/c mice (Taconic) were injected with 5 × 10^4^ luciferized MC26 cells in the subsplenic capsule to allow cells to seed the liver. After 90 seconds, the spleen was removed to prevent splenic tumors. 4–5-week-old female NCr nude mice (Taconic) were injected bilaterally with 2 × 10^6^ LS 174T cells per flank to generate subcutaneous xenografts, or implanted with 5 × 10^5^ D8–175 KPC cells to form flank pancreatic cancer allografts. Syngeneic subcutaneous grafts were generated by inoculation of 1 × 10^5^ MC26 cells in BALB/c mice. Tumor growth was monitored by luminescence on the In Vivo Imaging System (IVIS, PerkinElmer Inc.). Prior to induction of tumors, urine measurements were made by injecting nanosensors at 1 nmol (peptide substrate amount) per mouse. Post tumor inoculation urine measurements were performed when tumor luminescence reached an average of 1–1.5e7 photons/sec/cm^2^/sr. After injection with nanosensors, mice were placed into custom housing with a 96-well plate base for urine collection. The bladders were voided to collect between 100–200 μL of urine at 1 and 2 h post injection. Urine was centrifuged and stored at −20°C. For organ and tumor biodistribution and quantification, mice were sacrificed at 6 h or 24 h post nanosensor injection and organs were removed and scanned on the LI-COR Odyssey Infrared Imaging System with ImageStudio (PerkinElmer Inc.). Fluorescence from the peptides on nanoparticle scaffold was quantified using ImageJ software. For toxicity examination, 2 nmol of nanosensors (peptide substrate amount) were injected intravenously. After scarification, organs were fixed, paraffin-embedded, sectioned and stained with hematoxylin and eosin.

### Mouse treatments.

5-Fluorouracil (5-FU) was purchased (Sigma-Aldrich) and prepared as previously described.^47^ 5-FU or vehicle control (DMSO) was administered by intraperitoneal injection at in a 0.2 mL volume starting 1 week after cell injection for metastasis-initiation experiments. A 15 mg/kg dose per treatment was used for all experiments. Tumor-bearing mice were treated daily for 4 days, followed by once every other day until the end of the study. Experimental group sizes were practically associated according to the number of mice housed per cage (n=5 mice per cage). For treatment experiments, tumor inoculation cohorts were designed to have n=10 mice per group (one more cage as needed), from which n=5 animals were chosen to balance a similar range of tumor burden between the groups based on tumor signals quantified by IVIS to perform studies on. Tumor-bearing mice were closely monitored and euthanized following health-check advice given by an independent veterinary scientist responsible for animal welfare.

### Urine reporter analysis and *in vitro* protease activity assays.

The urinary reporter tagged with a Biotin/FAM ligand pair was quantified using custom sandwich ELISAs that were previously described.^48^ The ligand pair was recognized by a mouse anti-fluorescein antibody (GeneTex, GTX10257) and NeutrAvidin-HRP (Pierce). Protease activity assays were performed in a 384-well plate in duplicate in MMP-specific buffer (50 mM TRIS, pH 7.5, 10 mM CaCl_2_, 300 mM NaCl, 20 μM ZnCl_2_, 0.02% Brij-35, 1% BSA) with intramoleculary quenched peptides (1 μM) and recombinant proteases (12.5 nM) in 30 μL at 37 °C. All recombinant enzymes were purchased from Enzo Life Sciences, Inc. Fluorescence was measured at Ex/Em 485/535 nm using an Infinite 200 PRO Tecan plate reader with Tecan icontrol software. Tissue samples were homogenized in cold PBS and centrifuged before total protein quantification with the Pierce^™^ BCA Protein Assay Kit (Thermo Fisher Scientific). 5 μL of supernatant (1mg/mL total protein) was placed into 384 well plate with 25 μL FRET peptides (1 μM) in MMP-specific buffer. Marimastat (Sigma) was used at a final concentration of 50 μM.

### Radiolabeling of PRISM, PET-CT imaging and analysis.

^64^Cu in the format of Copper Chloride aqueous liquid was obtained from the Mallinckrodt Institute of Radiology (MIR) Cyclotron Facility, Washington University School of Medicine in St. Louis. The ^64^Cu stock was equilibrated in 1x PBS solution prior to adding to maleimide-NOTA-labeled PRISM or non-targeting sensors in 1x PBS. The labeling reaction was allowed to proceed at room temperature for 20 minutes and was loaded on an Amicon Ultra Centrifugal filter (MWCO 10 kD, MilliporeSigma). Free ^64^Cu was separate by two 10 min spins at 12, 000 × g, remaining activity in nanosensors was measured by a CRC^®^ −25R dose calibrator (Mirion Technologies, Inc.) and ^64^Cu-chelated nanosensors was adjusted to ~100 μCi in 200 μL saline per mouse for intravenous injection.

Animal PET-CT imaging was performed on the G8 PET-CT preclinical imaging system (PerkinElmer Inc.) with a G8 Acquisition Engine. In all experiments involving direct comparison of ^18^F-FDG and ^64^Cu-PRISM, animals were imaged with ^18^F-FDG first followed by a subsequent ^64^Cu-PRISM imaging two days after according to the availability of radioactive traces. Mice were fasted for 12 h prior to PET-CT imaging with ^18^F-FDG. Each animal was anesthetized with isoflurane (2.5% for induction, 1.5–2% for maintenance in a heated chamber for ^18^F-FDG scanning). After placement of a catheter in the lateral tail vein for probe administration, mice were positioned in the imaging shuttle and a bolus injection (mean ~120 μCi) was delivered. Static imaging was performed for 10 min (1 frame) for sham and tumor-bearing mice 1 h post injection of ^18^F-FDG or 6 h post injection of ^64^Cu-PRISM. A whole-body CT was obtained immediately after the PET acquisition. CT images were acquired over ~1 min (peak tube voltage, 50 kV; tube current, 200 μA). The PET data were reconstructed using a 3D maximum-likelihood expectation-maximization algorithm run over 60 iterations. Normalization, decay-correction, attenuation-correction and dead time-correction were applied to all PET data acquired in listmode. Reconstructed PET and CT data were quantitatively evaluated using VivoQuant software (inviCRO Inc.). Regions of interest (ROIs) were drawn on the CT images in all planes (coronal, sagittal and transverse). The radioactivity uptake values within CT-drawn organs were obtained from mean voxel values within the ROI and converted to percentage injected dose (ID) and then divided by mouse weight (g) to obtain an imaging ROI-derived percentage of the injected radioactive dose per gram of tissue (%ID/g). Size of the ROI was ~465 mm^3^ for the lungs and ~503 mm^3^ for the liver. The tumor (or tissue) to tissue (i.e. lung to liver) ratio was calculated by dividing the mean lung by the mean liver %ID/g values. To quantify the radioactivity in diseased tissues *ex vivo*, tissues were harvested after perfusion with PBS and counts were collected using a 2480 WIZARD2 gamma counter (Perkin Elmer Inc.). A small fraction of the prepared dose was run as a Count Standard along with the tissues in order to quantify the percent injected dose per sample. All samples were decay corrected to the Count Standard measurement time.

### Cryo-fluorescence tomography (CFT).

CFT is a molecular tissue imaging modality developed by EMIT imaging. Immediately following PET imaging, the mice were sacrificed and the whole animals were frozen through slowly submerge the animals in dry-ice colded hexanes. Animals were left in hexanes for 5–10 minutes to allow complete frozen and optimum cutting temperature (O.C.T.) compound block. Tissue sectioning and imaging were performed on the EMIT Xerra^™^ platform. To visualize the FAM fluorophore on PRISM, excitation was set at 470 nm with emission at 511/20 nm. Deck of 25 μm tissue slices were imaged by a VivID^™^ Multi resolution viewer and reconstructed in the VivoQuant software (inviCRO Inc.).

### Histology and immunohistochemistry.

Sectioning and staining were performed by the KI Histology Core. Organs were fixed in 4% paraformaldehyde (PFA) and stored at 4 °C prior to embedding into paraffin, sectioning, and staining. Slides were stained with primary antibodies in accordance with manufacturer instructions, followed by HRP secondaries, and were digitized using an 3D Histech P250 High Capacity Slide Scanner (Perkin Elmer Inc.). Image visualization and analysis was conducted with a CaseViewer software (3DHISTECH Ltd.) Multiple organ cancer and normal tissue microarrays were obtained from US Biomax, Inc. (Catalog number CO702b). The TMA slides were stained with anti-MMP9 antibody (Abcam ab38898; 1:500), anti-CAIX antibody (Abcam ab15086, 1:200), respectively. Blind expression scoring of cores was done by Dr. R.T. Bronson at the KI histology core.

### Confocal microscopy and immunofluorescence.

Cells were seeded in a Nunc^™^ Lab-Tek^™^ Chambered Coverglass system (Thermo Fisher Scientific) in physiological (7.4) or acidic pH (6.4), incubated with VivoTag645-labeled pHLIP-nanosensors, and imaged under a Zeiss LSM 700 inverted confocal scanning microscope with an excitation wavelength of 633 nm and emission filter at 660–710 nm. Acquired images were processed with the ZEN software (Carl Zeiss Microscopy, LLC). To track the localization of nanosensors *in vivo*, tumor tissues were extracted, briefly fixed in 2% PFA, placed in 30% sucrose overnight and embedded in O.C.T. compound prior to sectioning. O.C.T. embedded tissue sections were stained with anti-FITC antibody (GeneTx GTX19224,1: 1000), anti-MMP9 antibody (Abcam ab38898; 1:500) or anti-biotin antibody (Abcam ab201341, 1:200). Fluorescence labeled secondary antibodies (Invitrogen, 1 μg/mL) were applied according to the primaries for 30 min at room temperature. Colocalization of MMP9 immunofluorescence and PRISM-labeled acidic areas in tumor sections was analyzed in ImageJ and quantified for the percentage of overlap between markers in red and green channels using inForm software (Perkin Elmer Inc.). Image quantification included tissue segmentation to segment tumor and nontumor regions followed by cell segmentation by DAPI counterstain using built-in algorithms. Number of MMP9 positive and PRISM positive cells were quantified in at least three areas per tumor section from at least three animals. MMP9- and PRISM-positive cells were quantified with cell-based overlap and Pearson correlation coefficient analyses. Average percentage of negative, single and double positives cell in each channel were reported.

### Statistical and Reproducibility.

Statistical analyses were conducted in GraphPad Prism. Data were presented as means with standard deviation (SD) or standard error of the mean (SEM). To determine whether there are any statistically significant differences between the means of independent groups, two-tailed *t*-test was used for two groups of data (parametric), and analysis of variance (ANOVA) was used for multiple group comparisons. Precise sample sizes, statistical tests and reproducibility of experiments are specified in the figure legends.

## Supplementary Material

Supplement

Supplementary movie 1

Supplementary movie 2

## Data Availability

The Cancer Genome Atlas (TCGA) Research Network (http://cancergenome.nih.gov) and FireBrowse (http://firebrowse.org) are open access resources with enriched cancer genomics data. All data that support the findings of this study are available within the article and Supplementary Information or from the corresponding author (sbhatia@mit.edu) upon reasonable request.

## Code availability

The accompanying code can be found on GitHub (https://github.com/lhaomit/PK-model-for-PRISM). The code was run on MATLAB_R2018b. The model description is available in the Supplementary Information.
