# Supplementary material for: Microenvironment-triggered multimodal precision diagnostics
Source: Nat Mater. Author manuscript; Available in PMC 2025 Aug 12. (PMC12341765; doi:10.1038/s41563-021-01042-y)
Supplement: Supplement [file NIHMS2099752-supplement-Supplement.pdf]

Supplementary Information

Microenvironment-triggered multimodal precision diagnostics

Liangliang Hao[1,2], Nazanin Rohani[1], Renee T. Zhao[2], Emilia M. Pulver[2], Howard Mak[1], Olivia J. Kelada[4], Henry Ko[1,2], Heather E. Fleming[1,8], Frank B. Gertler[1,3], Sangeeta N. Bhatia[1, 2, 5-8]

[1]Koch Institute for Integrative Cancer Research, Massachusetts Institute of Technology, Cambridge, MA 02139, USA

[2]Institute for Medical Engineering and Science, Massachusetts Institute of Technology, Cambridge, MA 02139, USA

[3] Department of Biology, Massachusetts Institute of Technology, Cambridge, MA 02139, USA

[4] Preclinical Imaging, PerkinElmer Inc., Hopkinton, MA 01748, USA

[5]Department of Electrical Engineering and Computer Science, Massachusetts Institute of Technology, Cambridge, MA 02139, USA

[6]Department of Medicine, Brigham and Women's Hospital and Harvard Medical School, Boston, MA 02115, USA

[7]Broad Institute of Massachusetts Institute of Technology and Harvard, Cambridge, MA 02139, USA

[8]Howard Hughes Medical Institute, Cambridge, MA 02139, USA

Corresponding author: Sangeeta N. Bhatia.

Address: 500 Main Street, 76-453, Cambridge, MA 02142, USA

Phone: 617-253-0893

Fax: 617-324-0740

Email: sbhatia@mit.edu

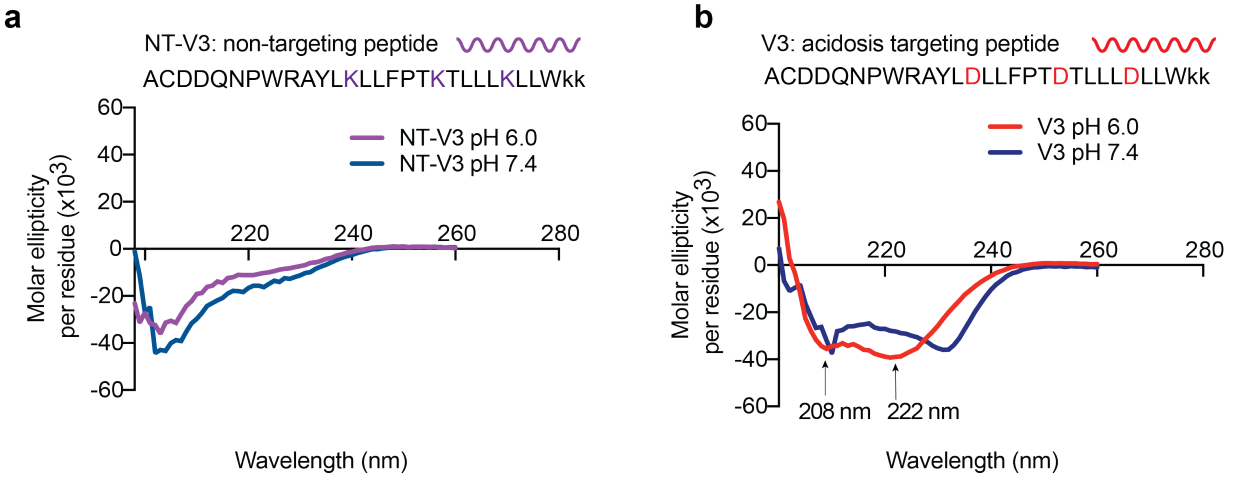

Supplementary Figure 1. Conformational characterization of acidosis targeting and non-targeting control peptides. Conformational changes of **a**, the non-targeting modification of pHLIP peptide (NT-V3) and **b**, the acidosis targeting pHLIP peptide (V3) were monitored under circular dichroism spectroscopy at physiological (pH=7.4) or pathological pHs (pH=6.0). Arrows: characteristic alpha-helix structure has negative bands at 208 nm and 222 nm. Amino acids that are critical for pH-dependent conformational switch and that are mutated in the non-targeting counterpart were highlighted.

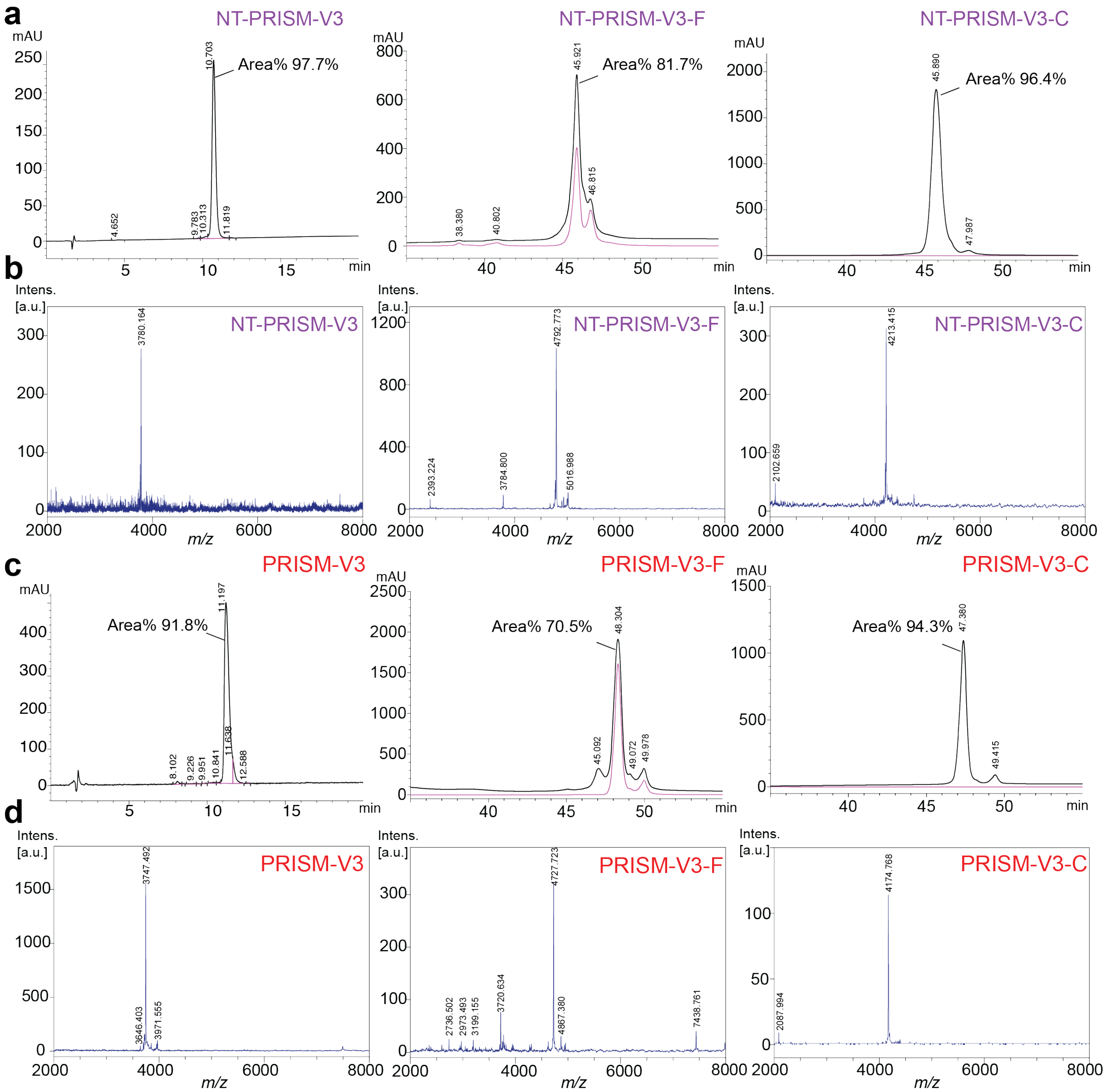

Supplementary Figure 2. Analytic characterization of acidosis targeting and non-targeting control peptide variants. a, RP-HPLC chromatogram of the non-targeting control peptide (NT-PRISM-V3) and its fluorophore (maleimide-VivoTag750, NT-PRISM-V3-F) or metal chelator (maleimide-NOTA, NT-PRISM-V3-C) conjugated variants. Purity of peptide or peptide conjugates corresponded to the area under the major peak in relation to the total area of all peaks detected within the chromatogram. Percentage of the major peak area of HPLC trace at 210 nm was indicated in each graph. Black curve, UV absorbance at 210 nm (ref 440 nm); magenta curve, absorbance at 700 nm (ref 440 nm). **b**, MALDI-TOF mass spectrometry analysis of peptide or peptide conjugates in **a**. Molecular weight was indicated on top of each peak. **c**, RP-HPLC chromatogram of acidosis targeting pHLIP peptide (PRISM-V3) and its fluorophore (maleimide-VivoTag 750, PRISM-V3-F) or metal chelator (maleimide-NOTA, PRISM-V3-C) conjugated variants. The area under the major peak corresponded to the percentage purity. The percentage peak area at 210 nm was indicated in each graph. Black curve, UV absorbance at 210 nm (ref 440 nm); magenta curve, absorbance at 700 nm (ref 440 nm). **d**, MALDI-TOF mass spectrometry for peptide or peptide conjugates in **c**. Molecular weight was indicated on top of each peak.

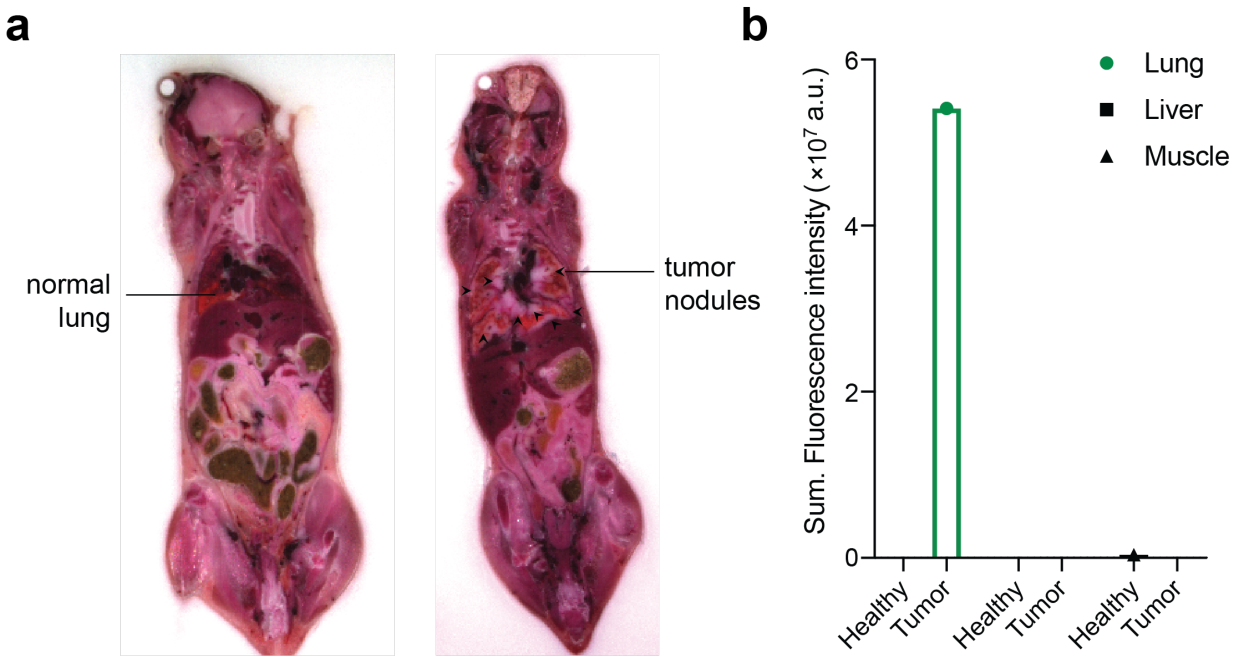

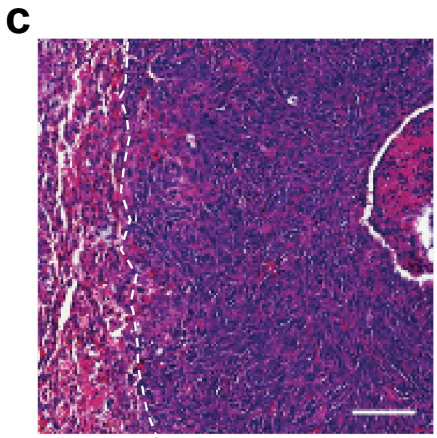

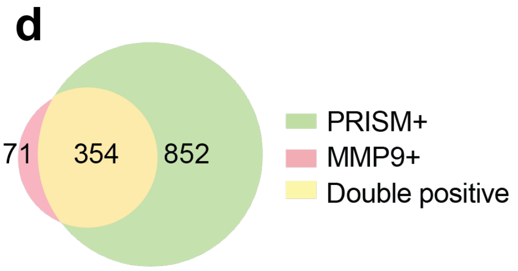

%cells	singles	doubles
PRISM	70.6	29.4
MMP9	16.9	83.1

Supplementary Figure 3. PRISM targeted invasive CRC lung nodules via tumor acidosis. a, Cryo-fluorescence tomography of healthy BALB/c mouse (left) and mouse with lung tumors (right) 6 h after PRISM administration. Images show the bright-and-white organ sectioning of the overlay of bright field and green fluorescence in **Fig. 1b**. **b**, Green fluorescence signal from PRISM in healthy or diseased lung tissues was quantified with VivoQuant software. **c**, Histological staining of image shown in **Fig. 1c**. Scale bar= 100 µm. Experiments were completed independently three times with similar results. **d**, Expression and colocalization analysis of MMP9 immunofluorescence (red) relative to PRISM-labeled acidic areas (green) in CRC lung lesion section shown in **Fig. 1c**. Venn diagram shows the average cell numbers and the table shows the percentage of single and double positive cells from each marker. Immunofluorescence staining was completed independently three times by different investigators with similar results.

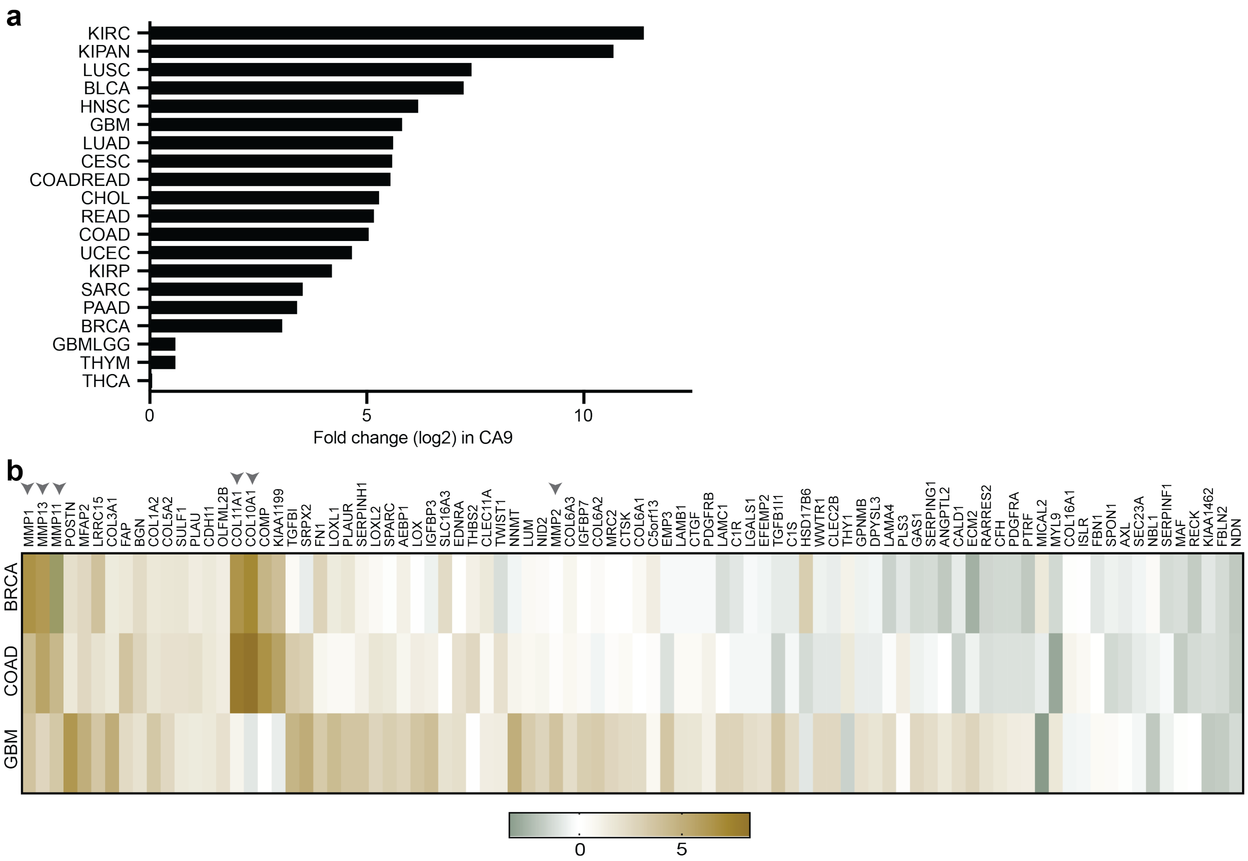

Supplementary Figure 4. Analysis of acidotic and proteolytic properties in human tumor microenvironment. a, Analysis of fold change of CA9 mRNA expression in numerous tumors in comparison with healthy controls compiled from TCGA data. **b**, Differential mRNA expression data analysis (TCGA) of multiple ECM regulators in adenocarcinomas and blastoma compared with normal adjacent tissue. Scale bar, fold change (log2) of differential gene expression. Arrowheads, highly expressed secreted collagen-degrading MMPs (i.e. MMP1, MMP11, MMP13) as well as ECM components that stimulate cancer cell invasion (i.e. COL10A1, COL11A1).

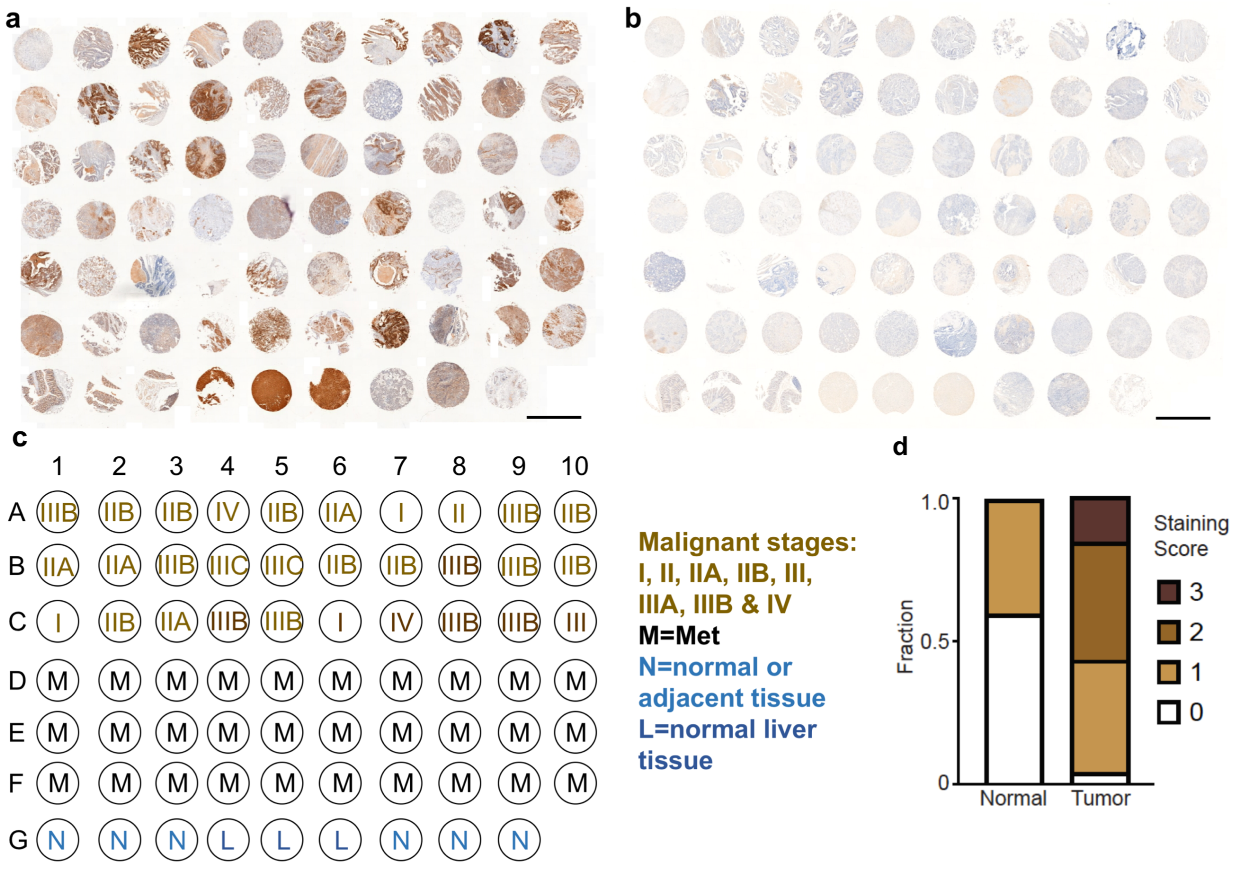

Supplementary Figure 5. IHC staining of tumor acidotic and proteolytic biomarkers in human Tissue Microarrays (TMAs). a, CA IX IHC staining of tumor samples, normal or adjacent tissues at different malignant stages (I, II, IIA, IIB, III, IIIA, IIIB and IV). **b**, MMP9 IHC staining of same samples as in (**a**). Scale bar= 2 mm. **c**, Map of TMA shown in **a** & **b**. Additional details can be found online at https://www.biomax.us/tissue-arrays/Colon/CO702b.**d**, Expression scoring of MMP9 protein levels in TMA of normal and COAD biopsies in **b**. Staining was completed twice with similar results.

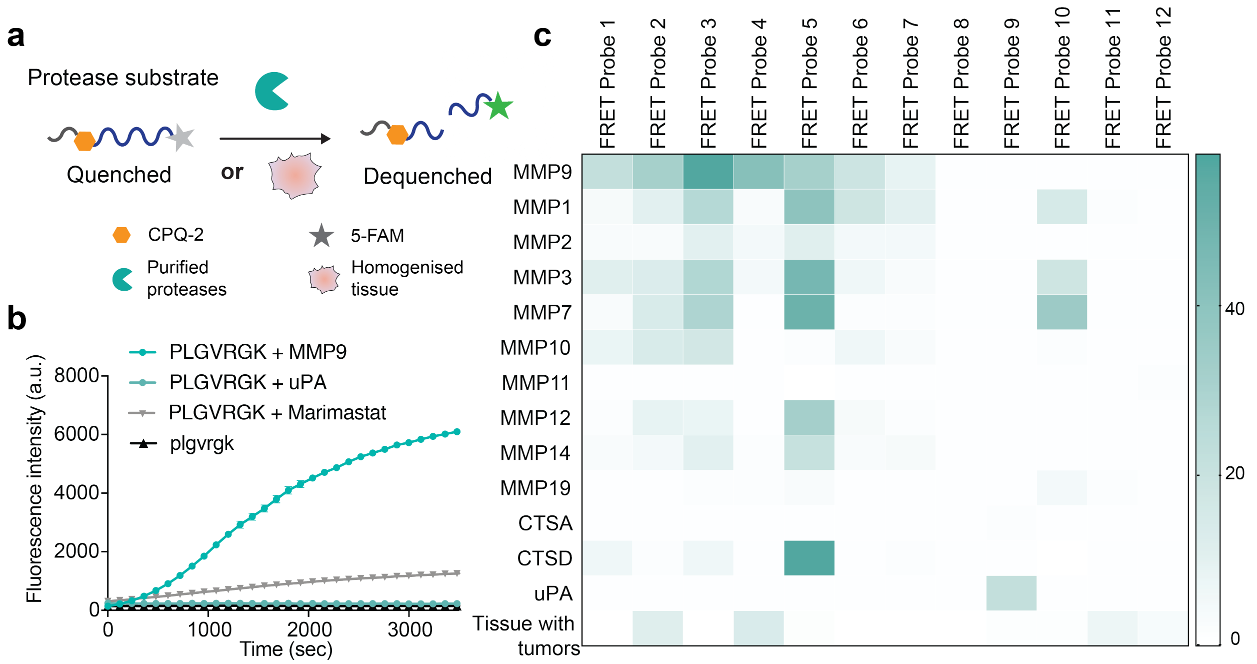

Supplementary Figure 6. *Ex vivo* screen of peptide substrate responsive for MMP9 cleavage. a, 12 FRET-paired protease substrates (Sequences in Supplementary Table 1), each consisting of a peptide sequence flanked by a FAM fluorophore and a CPQ-2 quencher, were screened against 10 recombinant matrix metalloproteinases (MMPs), 2 aspartic proteases (CTSA, CTSD) and 1 serine proteases (uPA). **b**, FRET signal was monitored by kinetic plate reader and kinetic fluorescence curves are shown for the FRET-paired substrates (PLGVRGK) with recombinant MMP9 and uPA. Control groups include assays with and without addition of MMP inhibitor Marimastat, and stabilized D-isomer substrate (plgvrgk). **c**, FRET-paired substrates were cleaved by recombinant enzymes or tissue homogenates of tissues from the BALB/c mice. The fold changes of FRET signal from all 12 FRET-paired substrates at 60 min after cleavage were subjected to heatmap. The bottom row represents the relative fold change of FRET signal from substrates cleaved by tissue homogenate of lung bearing CRC tumor nodules normalized against that of a normal lung tissue from BALB/c mice.

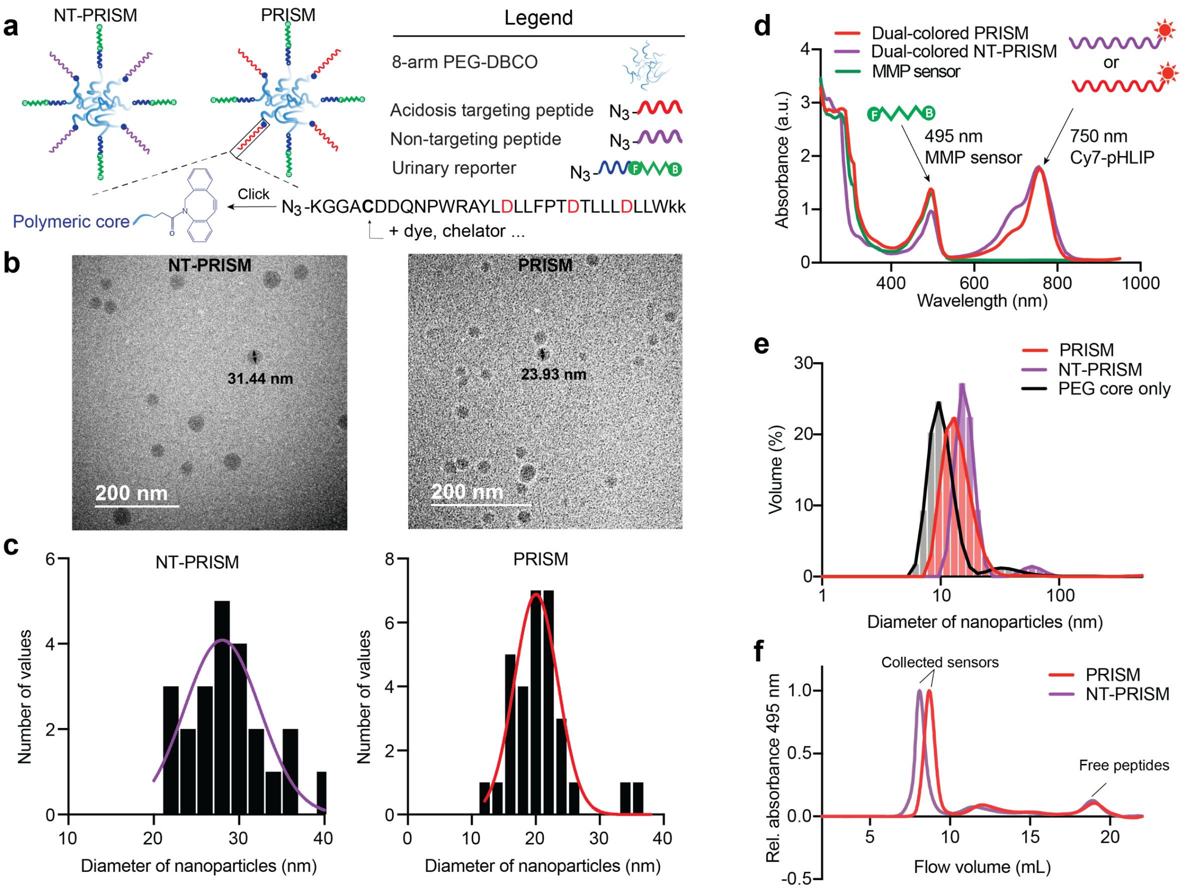

Supplementary Figure 7. Characterization of targeted and untargeted nanosensors. a, Schematic display on the composition and construction of non-targeting version of PRISM (NT-PRISM) and PRISM. Azide-terminated peptides were covalently linked to DBCO-containing polymeric core via copper-free click chemistry. Fluorescent dye or chelator was covalently attached to the single cysteine residue of acidosis targeting or non-targeting control peptide via maleimide-thiol reaction for optical or PET/CT imaging. **b**, Cryogenic electron microscopy (cryo-EM) of concentrated NT-PRISM (0.5 mg/mL) and PRISM (1 mg/mL). Representative images were taken at more than three locations of the sample grid. Experiments were completed twice independently. **c**, Histogram of nanoparticle size distribution by quantifying diameter of spherical particles in cryo-EM. **d**, Spectra of Cy7 and FAM dual-fluorescent PRISM, NT-PRISM and MMP sensor containing only FAM-labeled protease-activated reporter. FAM absorbs at wavelengths of 495 nm, targeting and non-targeting pHLIP tagged with Cy7 dye absorb at wavelengths of 750 nm. **e**, Size distribution of NT-PRISM, PRISM or plain polymeric core measured on the dynamic light scattering spectroscopy. **f**, Typical fast protein liquid chromatography (FPLC) chromatogram of NT-PRISM and PRISM from Superdex 75 10/300GL, showing the homogeneity of purified sensors. Detector wavelength was set at 495 nm.

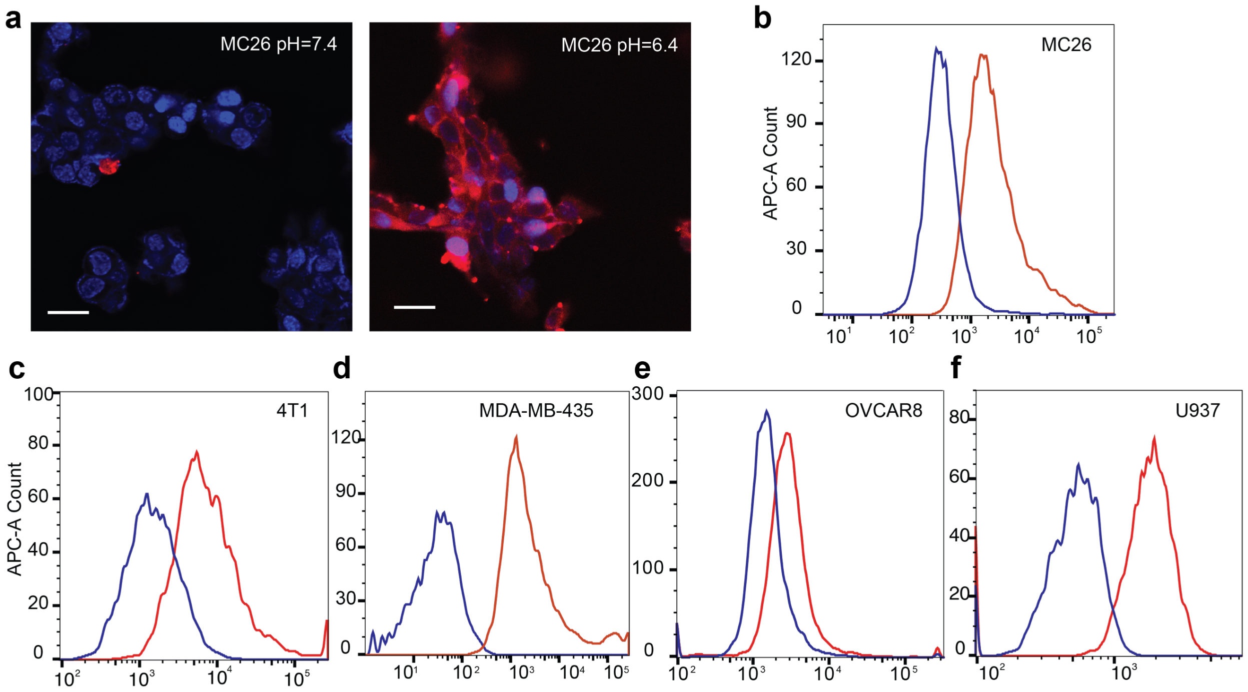

Supplementary Figure 8. PRISM targets multiple solid cancer cell lines. **a**, Confocal microscopy (Excitation 633 nm/Emission 650 nm) of MC26 cells incubated with fluorophore VivoTag645-labeled PRISM at pathological (pH 6.4) (left) and physiologic (pH 7.4) (right) conditions. Scale bar= 20 μm. **b-f**, Flow cytometry analysis of multiple cancer cell lines incubated with VivoTag645-labeled PRISM at physiologic (pH 7.4, blue) and pathological (pH 6.4, red) conditions. All experiments were completed independently three times with similar results.

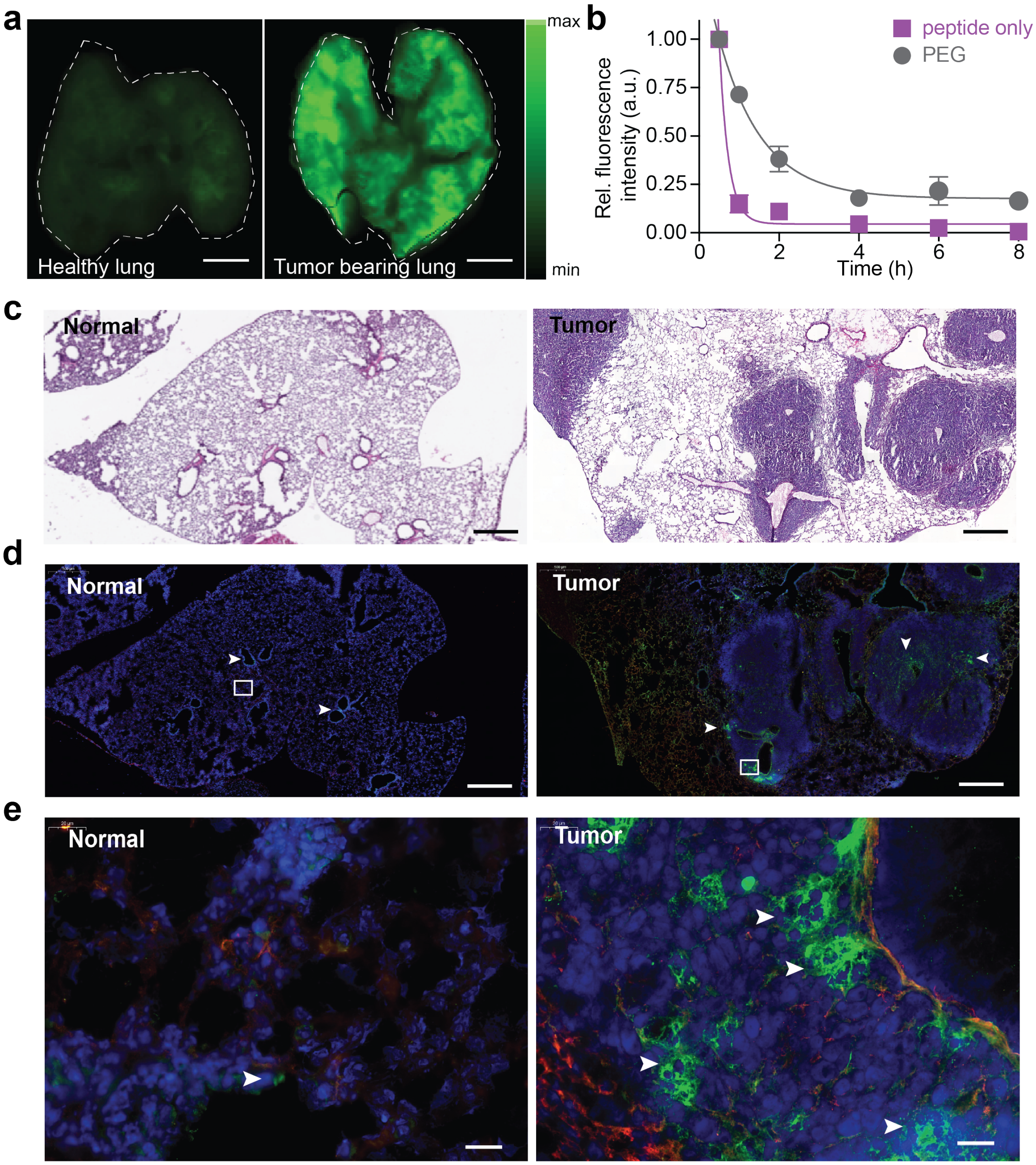

Supplementary Figure 9. PRISM enables efficient and safe tumor targeting in the CRC lung tumor model. a, Near-Infrared fluorescence imaging of lungs dissected from healthy (left) and BALB/c mice with CRC lung metastases (right) 6 hrs after i.v. injection of Cy7-labeled pHLIP. Tissues were scanned on an Odyssey CLx Fluorescence Imaging System (800 nm channel). Scale bar= 1 cm. **b**, Half-life of pHLIP peptide or peptide onto the PEG scaffold in plasma samples collected from healthy BALB/c animals after i.v. injection of peptide or that conjugated on a 8-arm, 40 kDa PEG scaffold (n=2 for the peptide only group, no error bars shown; n=3 for the PEG conjugate group; data are shown as means ± SEM). **c**, Representative histological images of lung from healthy and CRC lung tumor nodules bearing BALB/c mice at day 21 after tumor inoculation. Scale bar= 500 μm. **d**, Immunofluorescence staining of normal lung and tumor-bearing lung sections in (**c**) showing PRISM accumulation (green) and presence of MMP9 (red). Slides were scanned on a Pannoramic 250 Flash III whole slide scanner (3DHistech). **e**, Zoom-in images of (**d**), scale bar= 20 μm. Arrowheads, presence of PRISM accumulation (green). Experiments were

completed independently three times with similar results. Immunofluorescence staining was completed by different investigators.

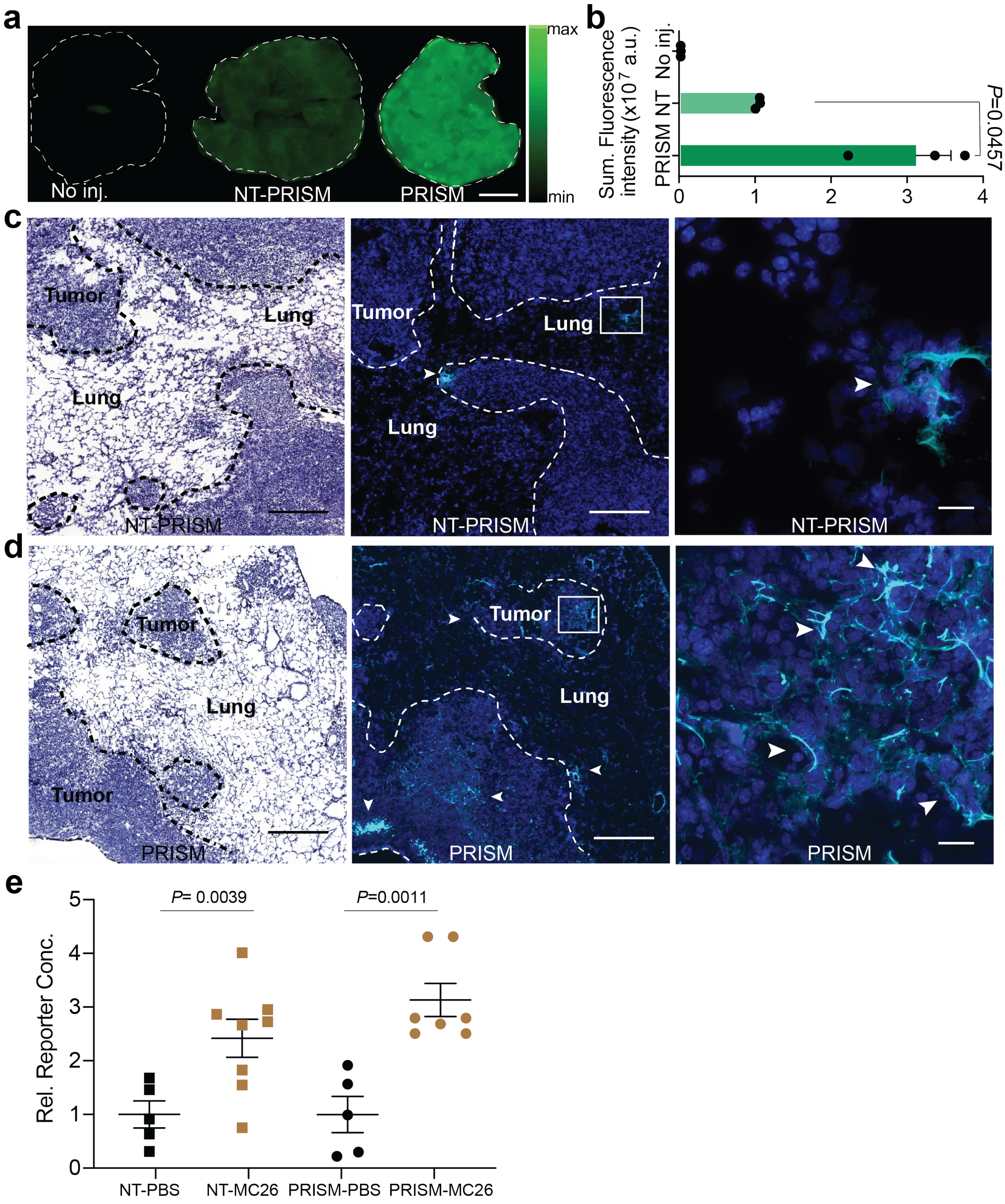

Supplementary Figure 10. PRISM localized the CRC lung nodules via acidosis targeting. a, Representative Near-Infrared fluorescence imaging of tumor-bearing mouse lungs 6 h after administration of VivoTag 750 (Excitation 750 nm, Emission 775 nm)-labeled NT-PRISM or PRISM. The maleimide-containing NIR dye was covalently linked to the cysteine residue of non-

targeting control peptide or pHLIP in NT-PRISM and PRISM. Tumor-bearing lungs collected from no injection control animals were scanned side-by-side on the Odyssey CLx Fluorescence Imaging System (800 nm channel). Scale bar= 1 cm. **b**, ImageJ quantification of NIR fluorescence in lung tissues scanned (n= 3 mice per group, data are shown as means ± SEM; unpaired two-tailed *t*-test with Welch's correction, *P<0.05). Tumor-bearing lungs shown in (**a**) were sectioned and histologically stained or directly imaged under the NIR channel of a Pannoramic 250 Flash III whole slide scanner (3DHistech) to visualize fluorescent signals from NIR dye-labeled NT-PRISM (**c**) or PRISM (**d**). Left, histological staining of tumor-bearing lung tissue sections. Scale bar= 500 µm. Middle, NIR fluorescence scan of the same region showing presence of NT-PRISM or PRISM (cyan). Scale bar= 500 µm. Right, zoom-in images of framed areas. Scale bar= 20 µm. Dotted lines, the edges of the tumor nodules and normal lung. Arrowheads, accumulation of NT-PRISM or PRISM (cyan). **e**, Relative reporter concentrations in the urine 1 h after NT-PRISM or PRISM injection in CRC lung tumor-bearing animals two weeks after tumor inoculation or age-matched healthy controls (n=5 mice per healthy control animal groups, n=7 mice per group for tumor-bearing group received PRISM injection, n=8 mice per group for tumor-bearing group received NT-PRISM injection, data are shown as means ± SEM; unpaired two-tailed *t*-test with Welch's correction). Experiments were completed independently twice with similar results.

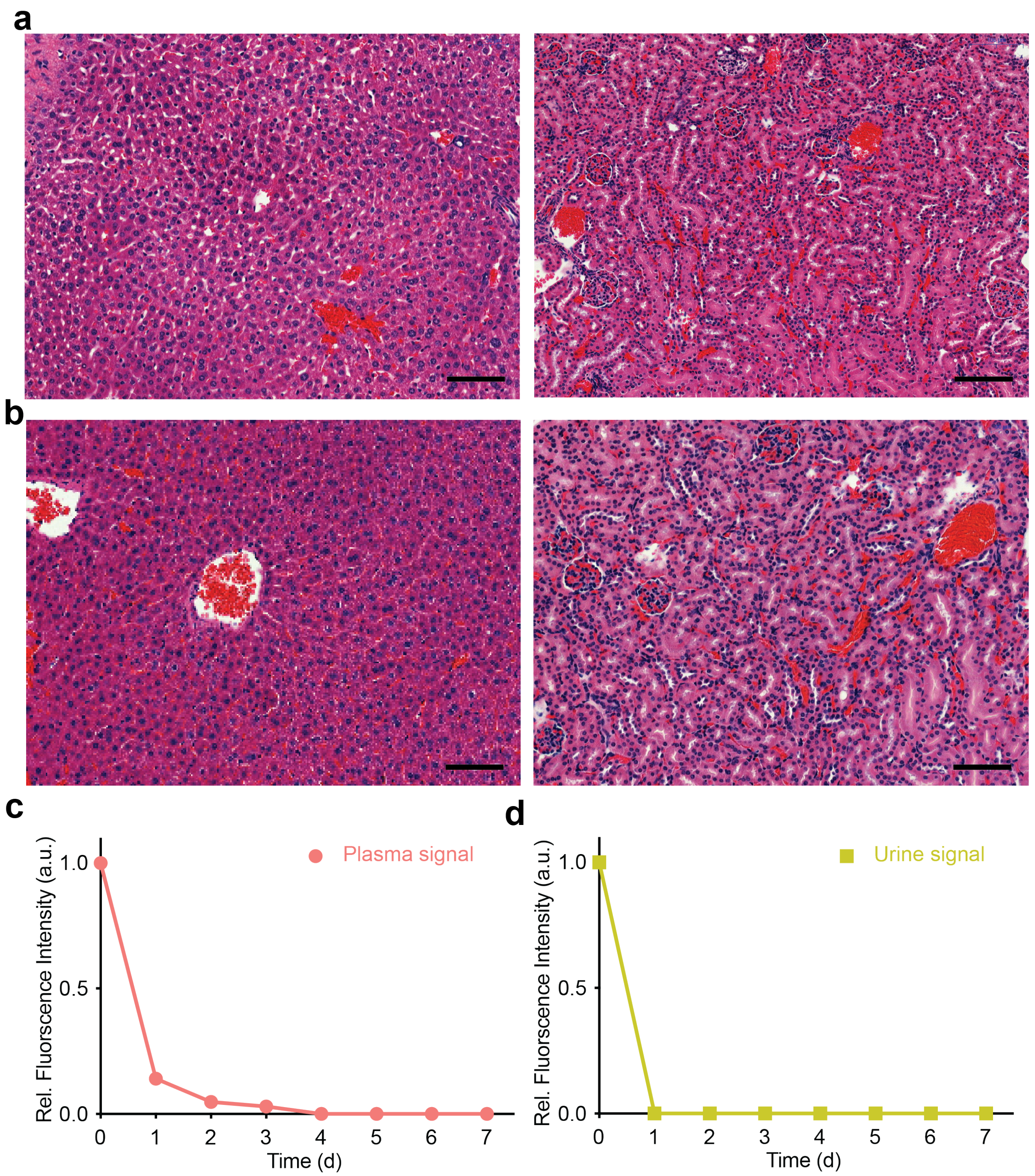

Supplementary Figure 11. Toxicity and clearance of PRISM *in vivo*. Representative histological images of livers (left) and kidneys (right) from healthy (**a**) and CRC lung nodule bearing BALB/c (**b**) mice after all urinary and imaging analyses at ~3 weeks of tumor inoculation. Scale bar= 100 µm. Experiments were completed independently three times with similar results. BALB/c mice were injected with PRISM intravenously and fluorescence was measured in (**c**) blood and (**d**) urine every 24 hours for 7 days.

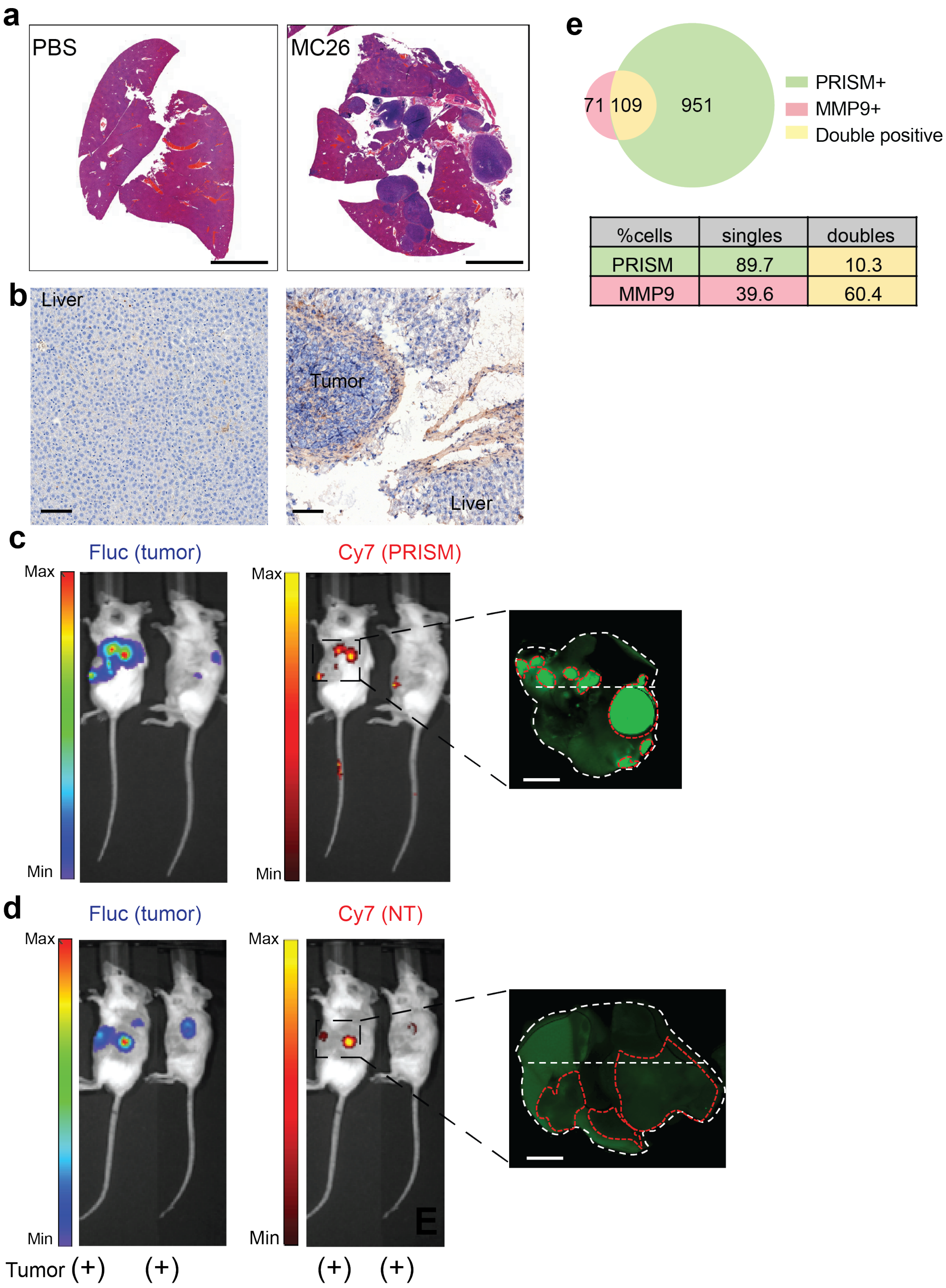

Supplementary Figure 12. PRISM enables visualization of CRC liver nodules in preclinical model. a, Representative histological images of livers from healthy and CRC liver nodules bearing BALB/c mice after 4 weeks of tumor inoculation. Scale bar= 500 μm. **b**, IHC staining of MMP9 in normal liver (left) and in liver with CRC liver metastasis (right). Positive stains are shown in brown. Scale bar= 100 μm. **c**, Cy7-labeled PRISMs and **d,** non-targeting sensors were i.v. administered in BALB/c mice bearing MC26 derived CRC liver metastases. Fluorescence imaging of Cy7-labled sensors (right) and luminescence imaging of luciferized tumor cells (left) were collected on IVIS. Livers from a representative animal in **c** & **e** were imaged on a Li-COR imager.

Organs were outlined in white. Scale bar= 1 cm. **e**, Expression and colocalization analysis of MMP9 immunofluorescence (red) relative to PRISM-labeled acidic areas (green) in CRC liver lesion section shown in **Fig. 3c**. Venn diagram shows the average cell numbers and the table shows the percentage of single and double positive cells from each marker.

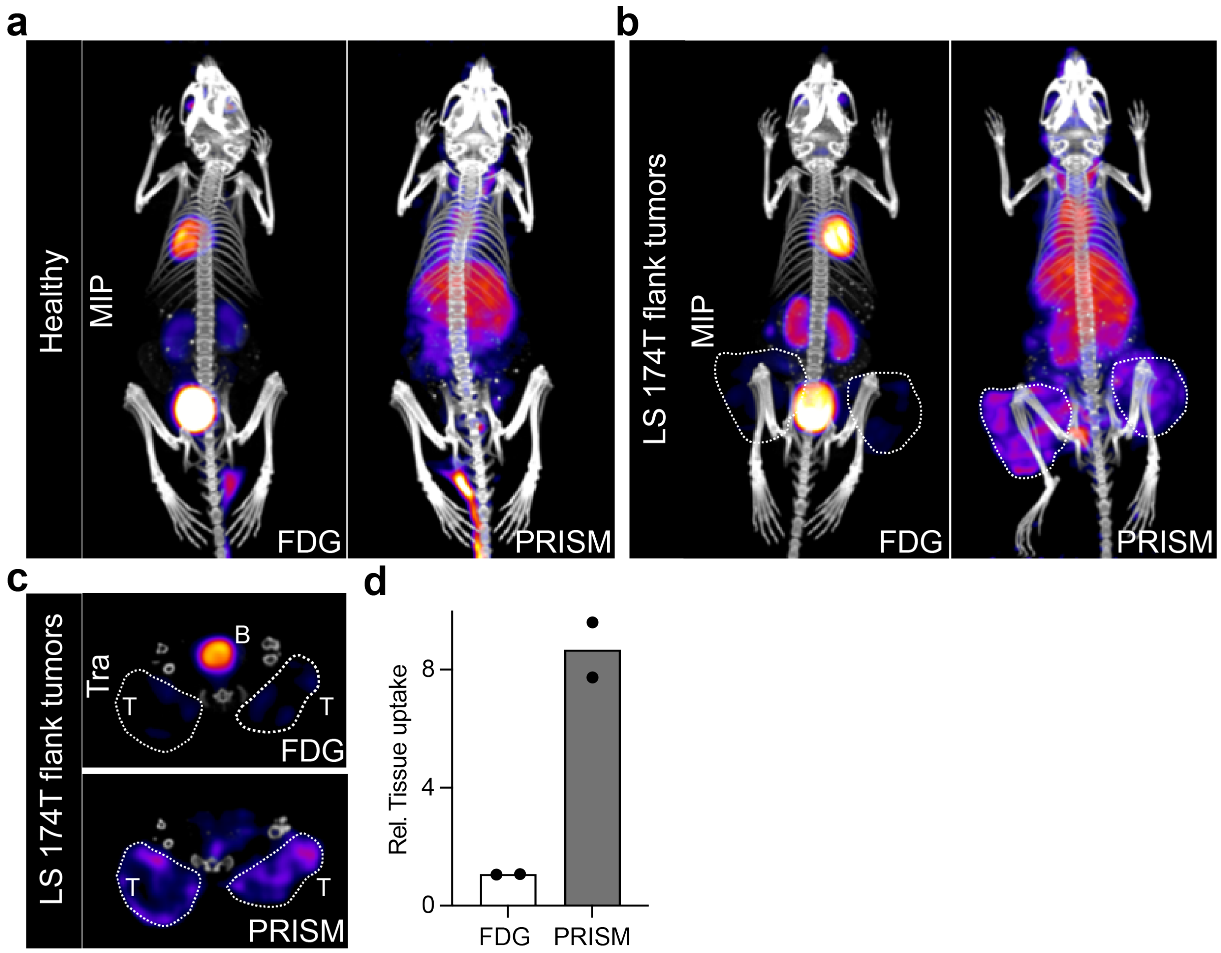

Supplementary Figure 13. PRISM enables FDG-negative tumor visualization. Representative PET images (maximum intensity projection, MIP) of whole healthy (**a**) or nude mice with subcutaneous tumors derived from low glucose uptake LS174T cell line (**b**) after i.v. injection of ^{64}Cu-PRISM and ^{18}F-FDG two days apart. **c**, Transverse view of subcutaneous LS174T tumors from the animal that was injected with ^{18}F-FDG or ^{64}Cu-PRISM, respectively. T, tumor; B, bladder. **d**, Quantification of the tissue uptake of ^{18}F-FDG and ^{64}Cu-PRISM in subcutaneous tumors (n=2 mice per group; data are shown as the ratio of tumor to surrounding normal tissue uptake in ID%/g; error bars are not shown for sample size n<3).

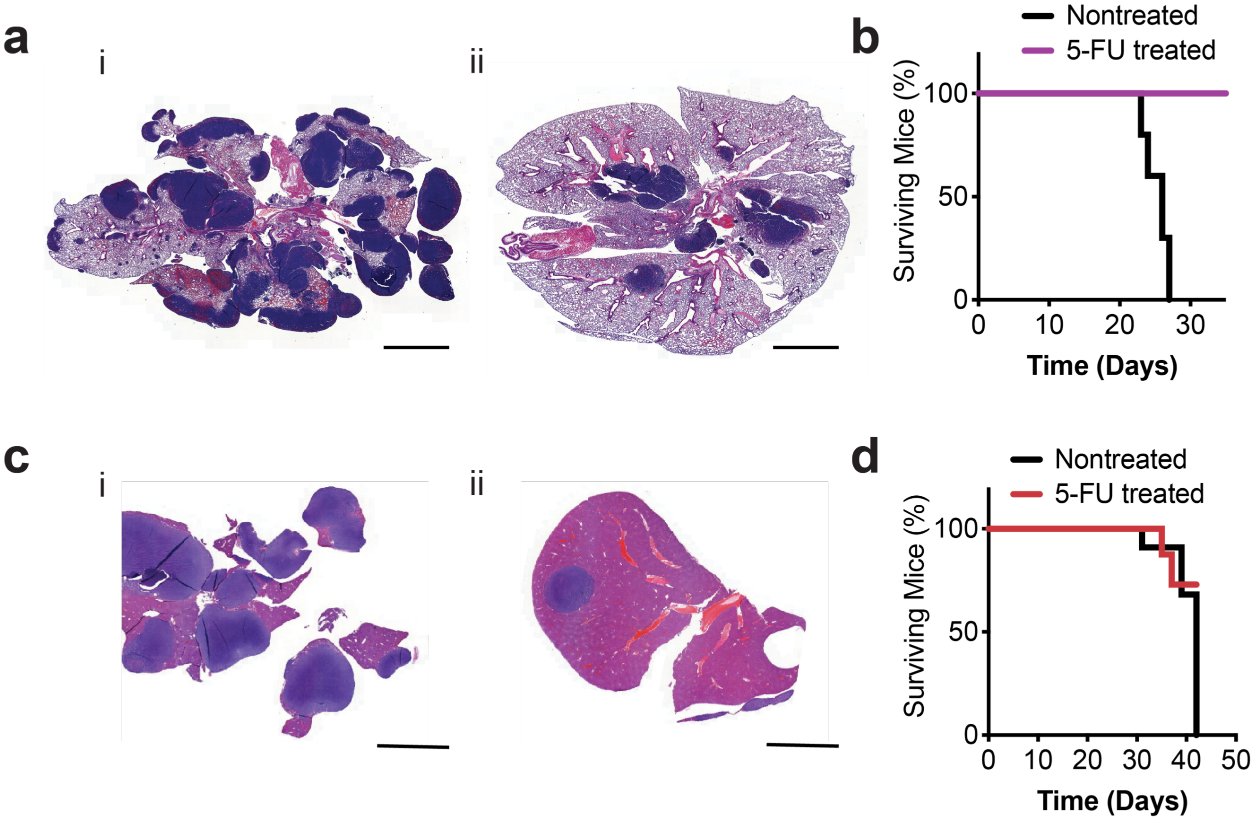

Supplementary Figure 14. Therapeutic responses to first line chemotherapeutic in preclinical models of metastatic CRC. a, H&E staining of the representative lungs dissected from untreated control (i) and 5-FU treated (ii) mouse sacrificed at 4 weeks after tumor inoculation. Scale bar= 2 mm. **b**, Survival curve of non-treated control and 5-FU treated cohorts of BALB/c mice bearing CRC lung metastases. **c**, H&E staining of the representative livers dissected from untreated control (i) and 5-FU treated (ii) mouse sacrificed at 7 weeks after tumor inoculation. Scale bar= 5 mm. **d**, Survival curve of untreated control and 5-FU treated cohorts of BALB/c mice bearing CRC liver metastases. Experiments were completed independently twice with similar results.

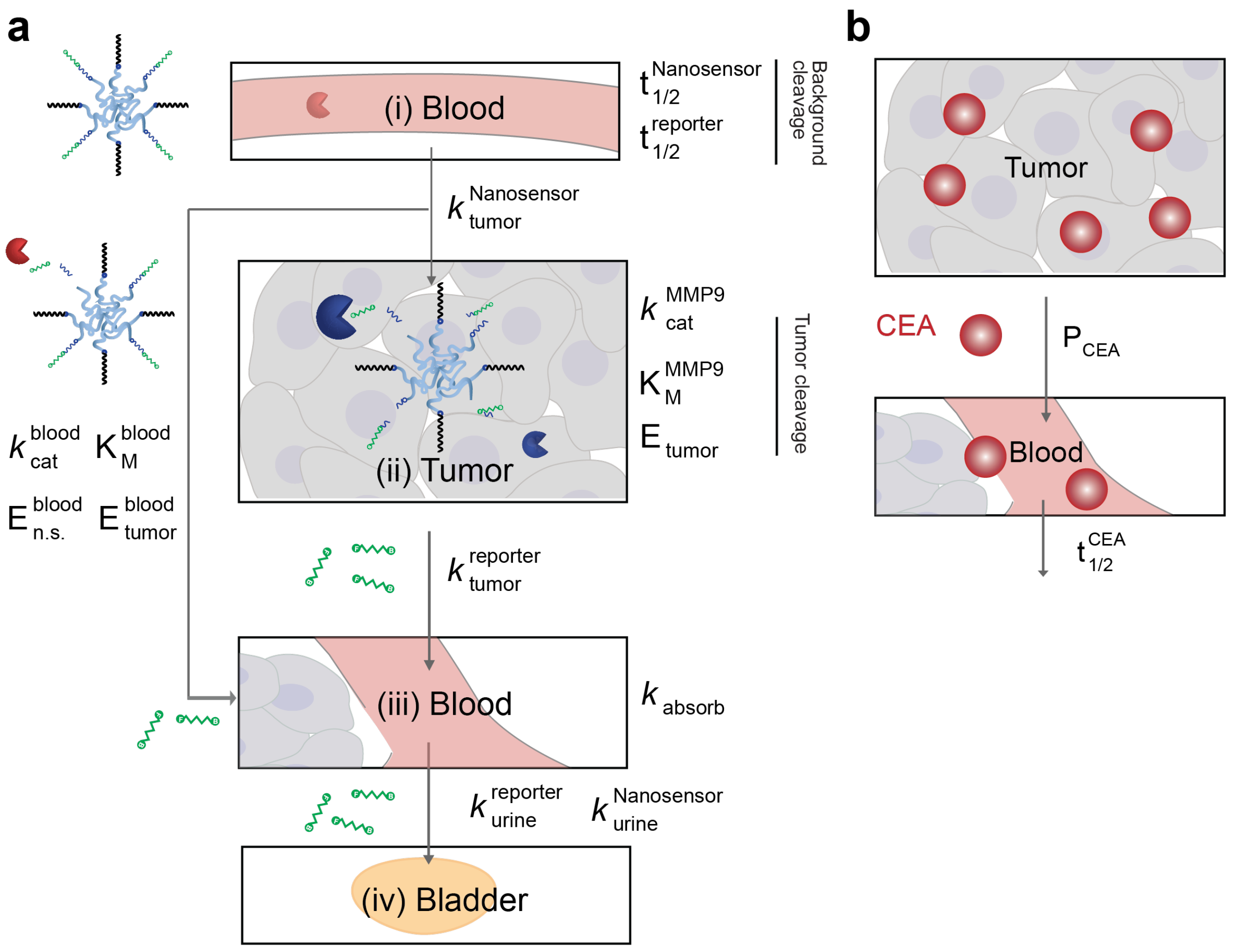

Supplementary Figure 15. Model-predicted behavior of activity-based nanosensors in CRC. **a**, Multicompartment model for activity-based nanosensors. In blood circulation (**compartment i**), intravenously administrated nanosensors diffuse into the tumor and are cleaved by tumor-associated proteases (e.g., MMP9) according to Michaelis−Menten kinetics (**compartment ii**). The cleaved reporters then diffuse back into blood (**compartment iii**), where they combine with reporters produced from nonspecific proteases and secreted tumor-associated proteases. Reporters from the blood accumulate in the bladder (**compartment iv**). Optimized activity-based nanosensors are obtained with eliminated background cleavage and enhanced tumor cleavage. **b**, Steady-state ODE model for the blood biomarker CEA.

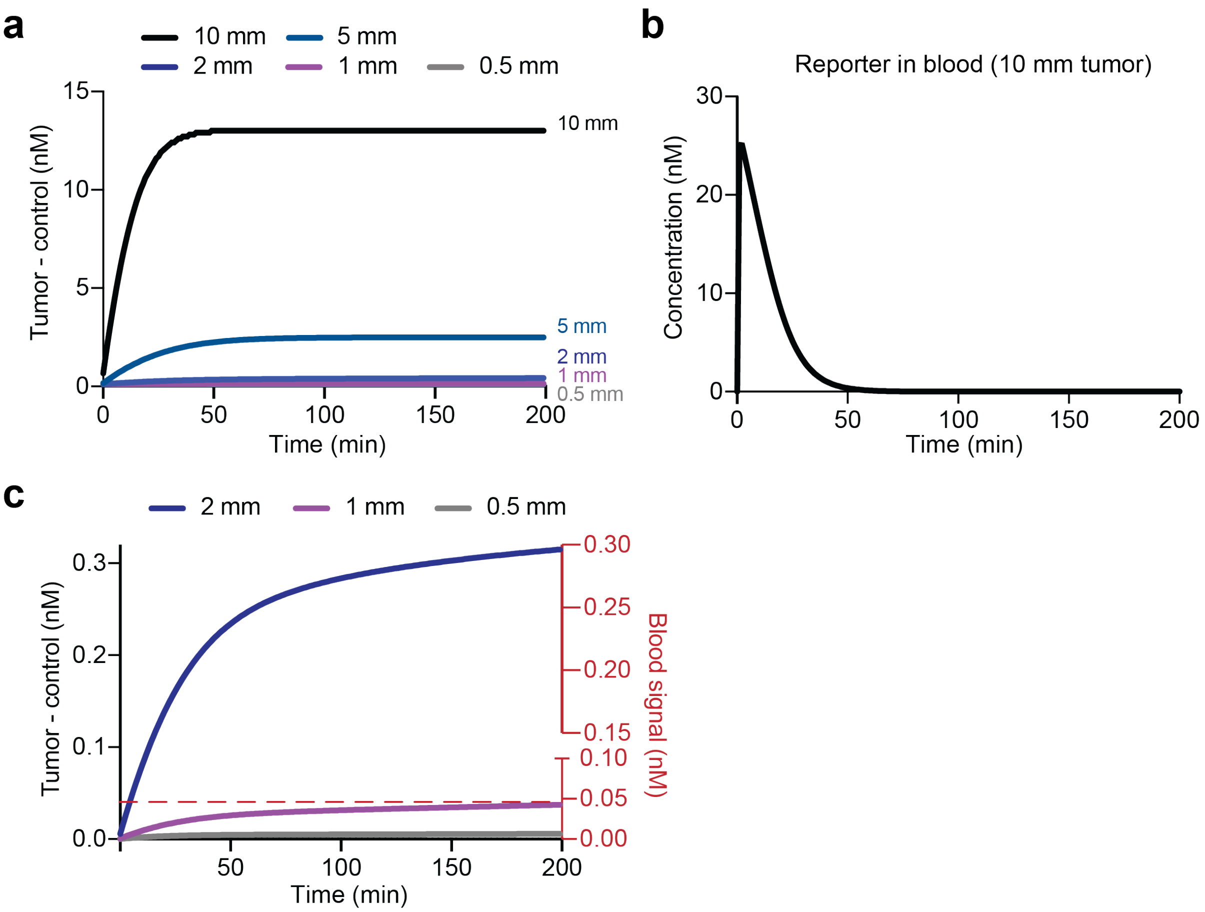

Supplementary Figure 16. Model-predicted behavior of targeted protease-activated nanosensors in CRC. **a**, Kinetic traces on change in urinary detection signal when optimized parameters are applied to different sizes (10, 5, 2, 1, and 0.5 mm) of tumors. **b**, Kinetics of plasma reporter in 10 mm tumors. **c**, Kinetic traces of detected urine signal in small sizes of tumor nodules (2, 1, and 0.5 mm) in comparison the blood biomarker CEA Steady-state ODE model (2 mm tumor; dotted red line).

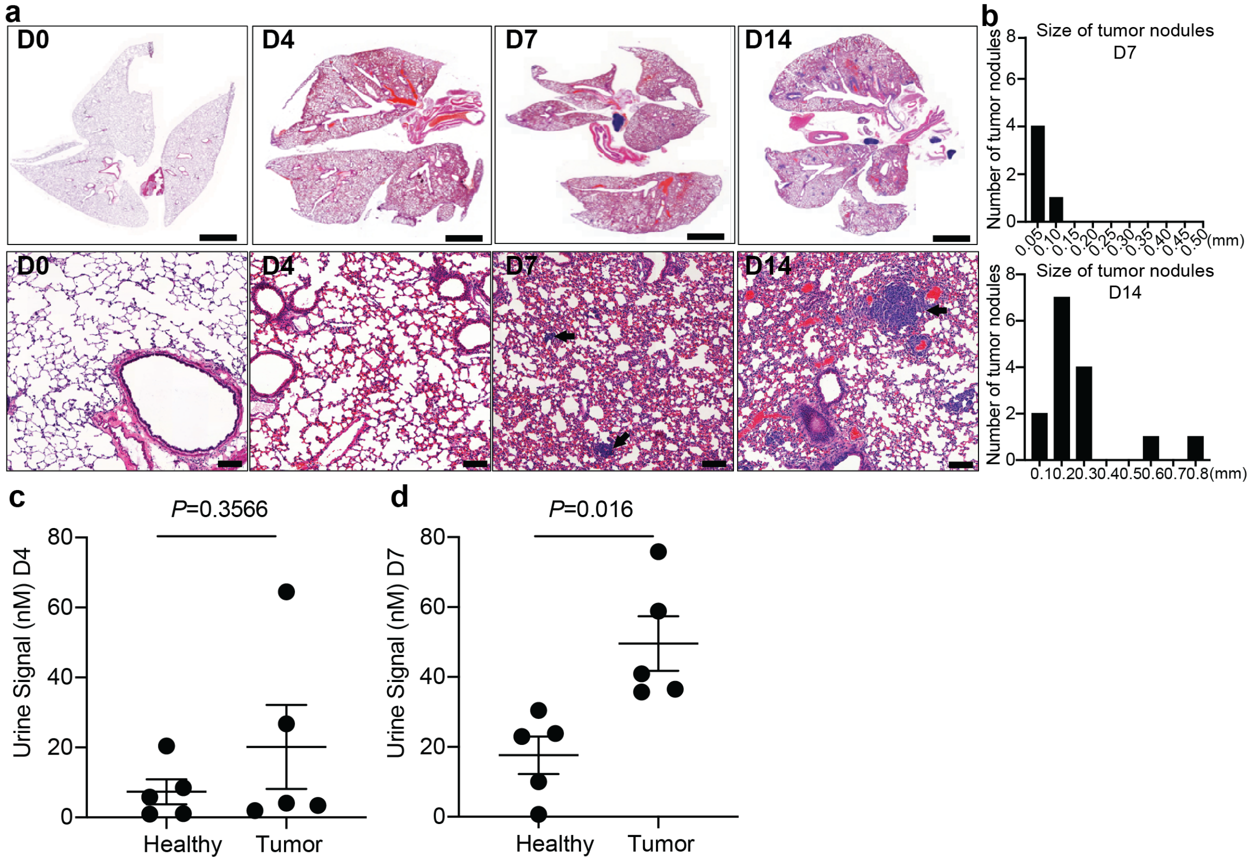

Supplementary Figure 17. Time course of PRISM validation in CRC lung tumor model. a, Representative histological images of lungs from CRC tumor-bearing BALB/c mice after day 0, 4, 7, 14 of tumor. Upper panel, scale bar= 2 mm. Lower panel, scale bar= 100 µm. Experiments were completed independently twice with similar results. **b**, numbers and sizes of tumor nodules counted in histological images in **a**. **c**, Relative reporter concentrations measured in the urine of healthy mice vs. CRC lung tumor-bearing mice after 4 days of tumor inoculation (n= 5 mice per group, data are shown as means ± SEM; unpaired two-tailed *t*-test with Welch's correction, not statistically significant). **d**, Relative reporter concentrations measured in the urine of healthy mice vs. CRC lung tumor-bearing mice after 7 days of tumor inoculation (n= 5 mice per group, data are represented as means ± SEM; unpaired two-tailed *t*-test with Welch's correction, *$P<0.05$).

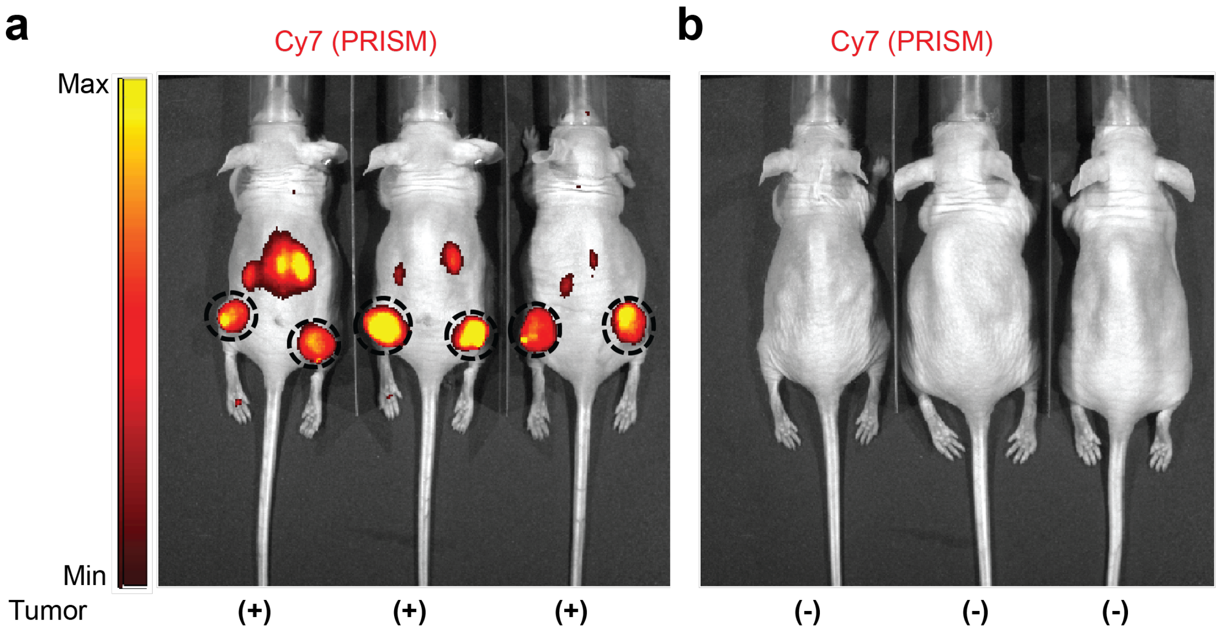

Supplementary Figure 18. Visualization of pancreatic cancer grafts with fluorescent PRISM. Fluorescence imaging of nude mice with subcutaneous bilateral flank grafts of pancreatic cancer (**a**) and healthy control mice (**b**) after 16 h after i.v. injection of Cy7-labeled PRISM. IVIS spectrum, Excitation 750 nm/ Emission 810 nm.

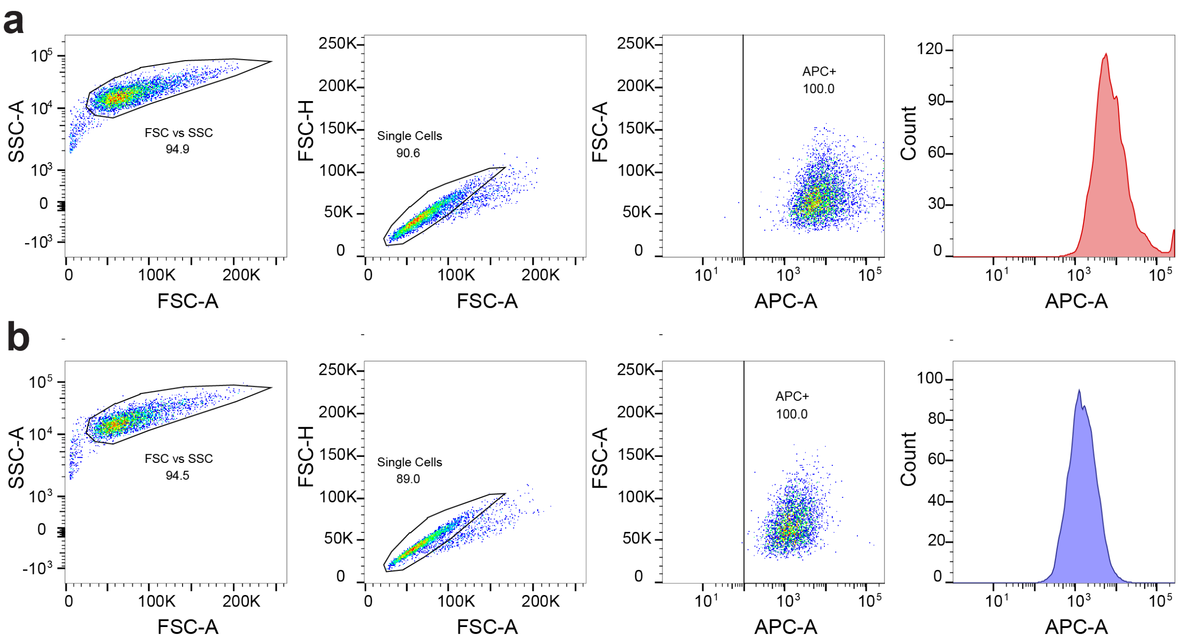

Supplementary Figure 19. Gating strategy for analyzing the fluorescence profiles of cultured cells on flow cytometer. Gating strategy was exemplified using 4T1 cells incubated with VivoTag645-labeled PRISM at (**a**) pathological (pH 6.4, red) and (**b**) physiologic (pH 7.4, blue) conditions. For all cell lines shown in Supplementary Figure 8, gates were set based on cell size and granularity (FSC-A vs SSC-A), singlets (FSC-H and FSC-A) and APC intensity. The cells analyzed in this work were defined as single cells and APC+. The signals of cells were measured in an LSR II HTS Flow Cytometer (BD Biosciences) and analyzed by FlowJo software. Each assay was completed independently for three times with similar results.

Supplementary Table 1. Peptides and peptide conjugates used in this study

Name of Peptide	Sequence (N→C)	MW (Da)	Purity
pHLIP-V3	ACDDQNPWRAYLDLLFPTDTLLLDLLWkk	3478.0	91.2%
pHLIP-V3-F	AC(Cy7)DDQNPWRAYLDLLFPTDTLLLDLLWkk	4282.9	93.7%
PRISM-V3-L	K(N3)GGACDDQNPWRAYLDLLFPTDTLLLDLLWkk	3746.3	91.8%
PRISM-V3-F	K(N3)GGAC(Cy7)DDQNPWRAYLDLLFPTDTLLLDLLWkk	4551.2	91.0%
PRISM-V3-F*	K(N3)GGAC(VivoTag750)DDQNPWRAYLDLLFPTDTLLLDLLWkk	4727.7	70.5%
PRISM-V3-C*	K(N3)GGAC(NOTA)DDQNPWRAYLDLLFPTDTLLLDLLWkk	4147.8	94.3%
NT-pHLIP-V3	ACDDQNPWRAYLKLLFPTKTLLLKLLWkk	3517.3	92.2%
NT-pHLIP-V3-F	AC(Cy7)DDQNPWRAYLKLLFPTKTLLLKLLWkk	4322.2	90.1%
NT-PRISM-V3-L	K(N3)GGACDDQNPWRAYLKLLFPTKTLLLKLLWkk	3785.6	97.7%
NT-PRISM-V3-F	K(N3)GGAC(Cy7)DDQNPWRAYLKLLFPTKTLLLKLLWkk	4590.4	93.4%
NT-PRISM-V3-F*	K(N3)GGAC(VivoTag750)DDQNPWRAYLKLLFPTKTLLLKLLWkk	4792.7	81.7%
NT-PRISM-V3-C*	K(N3)GGAC(NOTA)DDQNPWRAYLKLLFPTKTLLLKLLWkk	4213.4	96.4%
MMP9 substrate	Biotin-eGvndneeGffsarK(5FAM)-GGPLGVRGKGK(N3)	3316.6	91.8%
FRET Probe 1	5FAM-GGPVGLIGK(CPQ2)-PEG2-C	1895.0	91.6%
FRET Probe 2	5FAM-GGPVPLSLVMK(CPQ2)-PEG2-C	2195.5	92.6%
FRET Probe 3	5FAM-GGPLGLRSWK(CPQ2)-PEG2-C	2168.3	91.8%
FRET Probe 4	5FAM-GGPLGVRGKK(CPQ2)-PEG2-C	2066.2	95.7%
FRET Probe 5	5FAM-GGPVPLSLVMGK(CPQ2)-PEG2-GC	2507.9	90.9%
FRET Probe 6	5FAM-GGPWGIWGQGK(CPQ2)-PEG2-C	2240.4	91.8%
FRET Probe 7	5FAM-GGPQGIWGQK(CPQ2)-PEG2-C	2125.2	90.3%
FRET Probe 8	5FAM-GGLVPRGSGK(CPQ2)-PEG2-C	2025.1	91.2%
FRET Probe 9	5FAM-GGGSGRSANAKGK(CPQ2)-PEG2-GC	2300.4	94.2%
FRET Probe 10	5FAM-GILSRIVGGGK(CPQ2)-PEG2-GC	2338.6	90.7%
FRET Probe 11	5FAM-GSGSKIIGGGK(CPQ2)-PEG2-GC	2242.4	92.0%
FRET Probe 12	5FAM-GGf-Pip-RSGGGK(CPQ2)-PEG2-C	2031.1	93.5%
FRET Probe control	5FAM-GGplgvrgkK(CPQ2)-PEG2-C	2066.2	92.2%

Upper case, L-form amino acid;
lower case, D-form amino acid;
*, peptide conjugates labeled in-house;
N3, azide side chain;
5FAM, 5-Carboxyfluorescein; Cy7, Cyanine 7; VivoTag750, near-infrared fluorochrome.
Molecular weight of peptides was validated in mass spectrometry. The area under the major peak of HPLC traces corresponded to the percentage purity of peptides.

Supplementary Table 2. Example quantitative analysis of ^{64}Cu-PRISM PET-CT images in Fig. 2d.

Mouse	Lung (%ID/g)	Liver (%ID/g)	Lung/Liver Ratio	Increase vs Sham
Sham*	0.71	3.00	0.24	1
T0	1.26	1.45	0.87	3.68
T1	1.21	1.46	0.83	3.54
T2	1.37	1.47	0.93	3.95
T3	1.29	1.48	0.87	3.71
T4	1.88	1.96	0.96	4.06
T5	1.36	1.39	0.98	4.17

* One sham animal from a group of four healthy control mice is shown to demonstrate the normalization of Lung/Liver Ratio.

Supplementary Movie 1. Coronal view of cryo-fluorescence tomography in Fig. 1b.

Supplementary Movie 2. Sagittal view of cryo-fluorescence tomography in Fig. 1b.

Supplementary Note

Mathematical pharmacokinetic modeling

The original design of the pharmacokinetic model has been described previously (Kwong et al., 2015), where the model prediction data closely aligned with experimental observations. Descriptions, equations, and variables are derived from this reference and are included here with discussion of portions relevant for this work. The multicompartment model is comprised of five ordinary differential equations (ODEs) tracking the levels of nanosensor (protease substrate tethered onto a scaffold) and reporters (protease-cleaved fragments used for detection) in three compartments (Blood, Tumor, Bladder). The model is deterministic and has several simplifying assumptions. For example, it assumes a homogeneous, well-mixed distribution of nanosensors or reporters.

(1) Nanosensors (NS) circulate in the blood (i) after intravenous injection, they can be depleted by circulating non-specific (n.s.) proteases (k_{cat}^{blood}, K_M^{blood} $E_{n.s.}^{blood}$) in the blood or secreted proteases from the tumor (E_{MMP9}^{blood}), and passively diffuse into the tumor compartment (ii) by the rate constant k_{tumor}^{NS}. In the tumor, peptide substrate on the nanosensors are cleaved by tumor proteases, in this study, MMP9, following Michaelis−Menten enzyme kinetics (k_{cat}^{MMP9}, K_M^{MMP9}, E_{MMP9}^{tumor}). The cleaved fragments (reporters) passively diffuse to the blood compartment (k_{tumor}^{R})(iii), combined with reporters cleaved in the blood by circulating nonspecific protease, are taken by the host clearance system and filtered into urine (iv).

$$dC_{NS_{blood}} = -\left(k_{clear}^{NS}\right)*\left(C_{NS_{blood}}\right) - k_{tumor}^{NS}*\left(C_{NS_{blood}} - C_{NS_T}\right) - \frac{k_{cat}^{MMP9}*E_{MMP9}^{blood}*C_{NS_{blood}}}{\left(K_M^{MMP9}+C_{NS_{blood}}\right)} - \frac{k_{cat}^{blood}*E_{n.s.}^{blood}*C_{NS_{blood}}}{\left(K_M^{blood}+C_{NS_{blood}}\right)} \quad \textbf{(1)}$$

(2) At the tumor site, nanosensor concentration increases as they accumulate into the tumor and get depleted as they are cleaved by tumor proteases, here we use parameters of MMP9 for this model and assume negligible nonspecific protease activities within the tumor compartment.

$$dC_{NStumor} = k_{tumor}^{NS}*\left(C_{NS_{blood}} - C_{NS_T}\right) - \frac{k_{cat}^{MMP9}*E_{MMP9}^{tumor}*C_{NS_{tumor}}}{\left(K_M^{MMP9}+C_{NS_{tumor}}\right)} \quad \textbf{(2)}$$

(3) On-target proteolytic cleavage of the nanosensors releases reporters in the tumor that can diffuse back out of the tumor.

$$dC_{Rtumor} = \frac{k_{cat}^{MMP9}*E_{MMP9}^{tumor}*C_{NS_{tumor}}}{\left(K_M^{MMP9}+C_{NS_{tumor}}\right)} - k_{tumor}^{R}*\left(C_{R_{tumor}}\right) \quad \textbf{(3)}$$

(4) Reporters in plasma can be generated either by proteolytic cleavage in the tumor or by reporters entering from the tumor bed and depleted by clearance, urinary filtration, or non-specific absorption in the kidney. We assumed negligible nonspecific protease

activities within the tumor compartment, and here introduced a rate constant, k_{absorb}, to account for nonspecific binding (e.g., on blood vessels) and kidney reabsorption of the reporter.

$$dC_{R_{blood}} = \left(k^{R}_{tumor}\right)*\left(C_{R_{tumor}}\right) + \frac{k^{MMP9}_{cat} * E^{blood}_{MMP9} * C_{NS_{blood}}}{\left(K^{MMP9}_{M} + C_{NS_{blood}}\right)} + \frac{k^{blood}_{cat} * E^{blood}_{n.s.} * C_{NS_{blood}}}{\left(K^{blood}_{M} + C_{NS_{blood}}\right)} - \left(k^{R}_{clear}\right)*\left(C_{R_{blood}}\right) - \left(k^{R}_{absorb}\right)*\left(C_{R_{blood}}\right) \quad \textbf{(4)}$$

(5) Reporters from the blood (<2 kDa) are size-filtered by the kidney and concentrate in the bladder.

$$dC_{R_{bladder}} = \left(k^{R}_{filter}\right)*\left(C_{R_{blood}}\right) \quad \textbf{(5)}$$

All variables were referred to literature-reported values, and were fit to experimental data in biochemical assays of MMP9 and proteolysis of the selected substrate (PLGVRG), as well as in a xenograft model of colorectal cancer. The key variable we tested was the tumor enzyme concentration with different sized of tumors. Based on the measurements of MMP9 secretion rate of four human CRC cell lines, tumor enzyme concentration was set at 700 nM for a 10-mm tumor and 7 nM for a 5-mm tumor, and was estimated to be 0.45 nM for 2-mm, 56 pM for 1-mm and 7 pM for 0.5-mm tumors respectively. The concentration of MMP9 in blood is modeled as one-tenth of tumor concentration based on previous estimation of protein secretion into blood from tumors. The concentration of MMP9 in blood is modeled as one-tenth of tumor concentration based on previous estimations of protein secretion into blood from tumors. For non-tumor control urine signal, the tumor compartment and all associated variables are omitted.

We then compare the detection limitation of ABNs with conventional blood biomarkers; the mostly widely accepted carcinoembryonic antigen (CEA) in human CRC, has an established detection limit of 1-2 cm. To benchmark our urinary readout with blood biomarkers, we have adapted a steady-state ODE model for CEA (Kwong et al., 2015).

P_{CEA}= (Tumor cell density * CEA production rate * retention factor) / Volume of blood **(6)**

$$\frac{dCEA}{dt} = P_{CEA} - \alpha CEA = \frac{P_{CEA} t^{CEA}_{1/2}}{\ln(2)} \quad \textbf{(7)}$$

With the assumption of an average tumor cell density of 10^6 cells per cubic millimeter, a CEA retention rate of 90%, a half-life of 72 h, the minimum and maximum CEA production rates were calculated to be 15 pg and 260 ng per 10^6 cells per 10 d, respectively. Tumors smaller than 10 mm in diameter would result in CEA levels that are indistinguishable from the baseline threshold expected in healthy individuals (5 ng/mL). We next combined both models to analyze LS174T colorectal cancer xenograft model, which produce CEA at a rate 100-fold above the median of 24 human CRC cell lines (median value = 1.5 ng per 10^6 cells per 10 d). Our model predicted that our activity-based probe would result in urine signals that would eclipse the steady-state level of CEA in blood at as small as 2-mm-size.

Parameters used in the model

Parameters are adopted from the original model (Kwong *et al.*, 2015) unless otherwise specified or referenced.

	Parameter	Description	Unit	Value
Tumor-specific protease activity (MMP9)	k_{cat}^{MMP9}	MMP9 cleavage kcat for substrate	min-1	0.5
	K_{M}^{MMP9}	MMP9 cleavage *Km*	M	2.13e-06
	E_{MMP9}^{tumor}	concentration of proteases in tumor	M	7.1624e-12~ 7.1624e-7
Nonspecific plasma enzyme kinetic constants	k_{cat}^{blood}	Plasma cleavage Kcat for MMP9 substrate	min-1	0.0659
	K_{M}^{blood}	Plasma cleavage Km	M	1.0063e-05
	E_{MMP9}^{blood}	concentration of shed proteases in blood	M	7.1624e-8
		concentration in blood	M	4e-6
Nanosensor and reporter clearing constants	k_{urine}^{NS}	Urinary filtration rate of nano- sensor	min-1	1.7e-05
	k_{tumor}^{R}	Urinary filtration rate of reporter	min-1	0.032
	k_{absorb}	Sensor and reporter reabsorption	min-1	2.89
Tumor permeability	k_{cat}^{MMP9}	rate of NP diffusion across tumor vessels	min-1	1.4e-04[17]
	K_{M}^{MMP9}	rate of reporter diffusion across tumor vessels	min-1	0.09
CEA		Plasma cleavage CEA secretion rate	ng/cells/day	0.015-270
	$t_{1/2}^{CEA}$	Plasma half-like of CEA	h	72
